# Factor inhibiting HIF can catalyze two asparaginyl hydroxylations in VNVN motifs of ankyrin fold proteins

**DOI:** 10.1016/j.jbc.2022.102020

**Published:** 2022-05-07

**Authors:** Thomas M. Leissing, Adam P. Hardy, Hokfung Chan, Yihua Wang, Anthony Tumber, Rasheduzzaman Chowdhury, Tianshu Feng, Mathew L. Coleman, Matthew E. Cockman, Holger B. Kramer, Georgina Berridge, Roman Fischer, Benedikt M. Kessler, Peter J. Ratcliffe, Xin Lu, Christopher J. Schofield

**Affiliations:** 1 Chemistry Research Laboratory, Department of Chemistry and the Ineos Oxford Institute for Antimicrobial Research, University of Oxford, Oxford, United Kingdom; 2 Ludwig Institute for Cancer Research, Nuffield Department of Clinical Medicine, University of Oxford, Oxford, United Kingdom; 3 NDM Research Building, University of Oxford, Oxford, United Kingdom; 4 Institute of Cancer and Genomic Sciences, University of Birmingham, Birmingham, United Kingdom; 5 The Francis Crick Institute, Ratcliffe Laboratory, London, United Kingdom; 6 MRC London Institute of Medical Sciences, London, United Kingdom; 7 Institute of Clinical Sciences, Imperial College London, Hammersmith Hospital Campus, London, United Kingdom

**Keywords:** ankyrin, hypoxia-inducible factor, factor inhibiting HIF, iron and 2-oxoglutarate/alpha-ketoglutarate oxygenase, JmjC demethylase, post-translational modification/hydroxylation, epigenetics, ARD, ankyrin repeat domain, ASPP, apoptosis stimulating of p53 protein, FIH, factor inhibiting hypoxia-inducible factor, GST, glutathione-*S*-transferase, HA, hemagglutinin, HIF, hypoxia-inducible factor, MS, mass spectrometry, 2OG, 2-oxoglutarate, SPE, solid phase extraction

## Abstract

The aspariginyl hydroxylase human factor inhibiting hypoxia-inducible factor (FIH) is an important regulator of the transcriptional activity of hypoxia-inducible factor. FIH also catalyzes the hydroxylation of asparaginyl and other residues in ankyrin repeat domain–containing proteins, including apoptosis stimulating of p53 protein (ASPP) family members. ASPP2 is reported to undergo a single FIH-catalyzed hydroxylation at Asn-986. We report biochemical and crystallographic evidence showing that FIH catalyzes the unprecedented post-translational hydroxylation of both asparaginyl residues in “VNVN” and related motifs of ankyrin repeat domains in ASPPs (*i.e.*, ASPP1, ASPP2, and iASPP) and the related ASB11 and p18-INK4C proteins. Our biochemical results extend the substrate scope of FIH catalysis and may have implications for its biological roles, including in the hypoxic response and ASPP family function.

2P-Oxoglutarate (2OG)-dependent oxygenases play central roles in the responses of animals to hypoxia. When sufficient dioxygen is present, efficient prolyl-4-hydroxylation of hypoxia-inducible factor alpha (HIF-α) subunits (as catalyzed by PHD or EGLN isoforms) signals for HIF-α degradation by promoting its binding to a von Hippel-Lindau protein ubiquitin ligase complex, so reducing the concentration of transcriptionally active heterodimeric α,β-HIF and HIF mediated transcription (1, 2, 3). When dioxygen levels are reduced, PHD catalysis is decreased, with consequent increases in HIF-α and α,β-HIF levels, leading to transcription of HIF target genes, for example, those encoding for erythropoietin and vascular endothelial growth factor, which act to counter the effects of hypoxia (4, 5, 6). At least in higher animals, a second oxygenase, factor inhibiting HIF (FIH), which like the PHDs is Fe(II) and 2OG dependent, regulates the activity of HIF-1α/2α isoforms by catalyzing the C-3 hydroxylation of a single asparagine residue in the HIF-1α/2α transcriptional activation domain, a modification that decreases binding of HIF with the CBP/p300 histone acetyltransferases, which are activators of transcription (7, 8, 9, 10).

In addition to HIF-α, FIH also interacts with multiple ankyrin repeat domain (ARD)–containing proteins, many, but not all, of which it hydroxylates, in a manner regulated by the ARD sequence and factors including the overall ARD fold (11, 12, 13, 14, 15). Rather unexpectedly, given crystallographic analyses on FIH–HIF-1α CAD fragment complexes, which suggested rather precise binding of the asparaginyl-substrate residue at the active site (16), FIH also catalyzes hydroxylation of aspartyl residues, histidinyl residues, and other residues in ARDs, often apparently within a preferred recognition sequence (-LLxxGADV**N**A-, with the hydroxylation site in bold) (17, 18).

By contrast with the apparent relatively “switch-like” role of FIH-catalyzed asparaginyl hydroxylation in the HIF system, the role(s) of FIH-catalyzed ARD hydroxylation is unclear. ARD hydroxylation can thermodynamically stabilize isolated ARDs (19, 20), but the physiological relevance of this is unclear. Variations in the extent of ARD hydroxylation and lifetimes of ARDs coupled with the observation that hydroxylated ARDs bind less tightly to FIH have led to the proposal that ARD hydroxylation is a potential mechanism for establishing “hypoxic memory” (12, 21, 22).

Janke *et al.* (23) have reported that FIH catalyzes asparaginyl hydroxylation of the ARD-containing apoptosis stimulating of p53 protein (ASPP) family members; ASPP2 was reported to undergo monohydroxylation of Asn-986. Here, we report biochemical and biophysical studies on the extent of FIH-catalyzed ASPP family hydroxylation; notably, the results reveal an unprecedented double asparaginyl hydroxylation of the “VNVN” motif present in the ARDs of ASPP1 and ASPP2.

## Results

### Initial cell evidence for VNVN double hydroxylation

**Figure 1:**
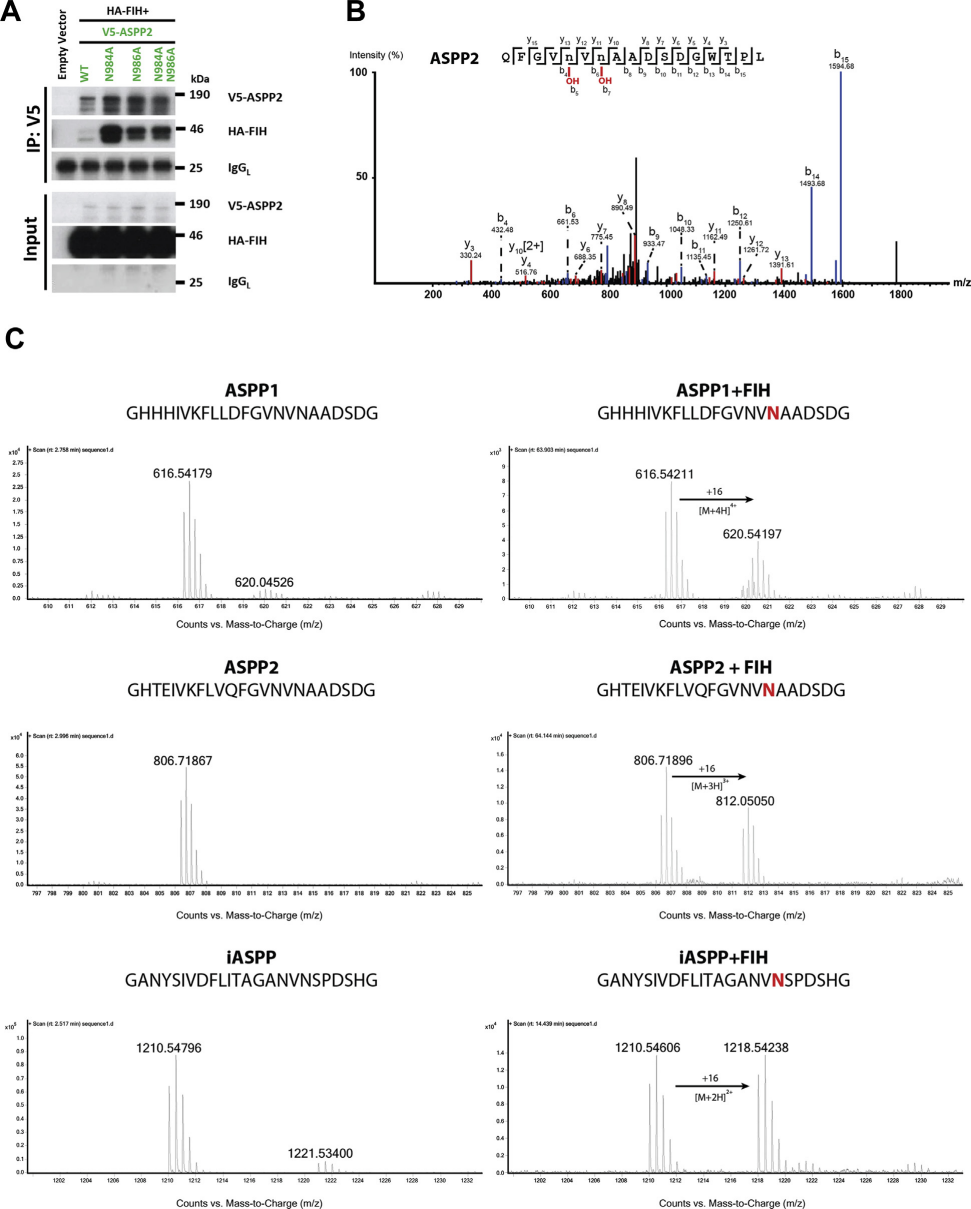
**Evidence for FIH-catalyzed double hydroxylation of ASPP2-V5 within the same ankyrin repeat in protein from U2OS cells but not in studies with peptide fragments.***A*, Western Blot analysis of V5-ASPP2 variants and HA-FIH using total cell lysates from ASPP2/FIH-double KO U2OS cells, transfected with an empty vector, or cotransfected with pcDNA3 vectors encoding for HA-tagged FIH and V5-tagged WT ASPP2 or “NVN” variants, immunoprecipitated (IP) with an anti-V5 antibody (Thermo Fisher Scientific). HA-tagged FIH, V5-ASPP2, and IgG light-chain (IgG_L_) levels are indicated. *B*, LC–MS/MS analysis of V5-ASPP2 from U2OS FIH CRISPR KO cells cotransfected with vectors encoding for V5-ASPP2 and HA-FIH. The spectrum shows fragments from the elastase-catalyzed digestion of ASPP2 showing evidence for hydroxylation at Asp-984 and Asp-986 (indicated by lowercase n). Conditions: V5-ASPP2 in pcDNA3. The vectors encoding for ASPP2 and FIH variants were transfected and overexpressed for 24 h in U2OS FIH CRISPR KO cells. Immunoprecipitation was employed with an anti-V5 antibody. Proteins were separated by SDS-PAGE; the band corresponding to ASPP2 was excised and digested using elastase for 16 h at 37 °C. *C*, LC–MS-based hydroxylation assays of an ASPP1-derived peptide (residues 932–954), an ASPP2-derived peptide (residues 969–991), and an iASPP-derived peptide (residues 670–693). Conditions: 0.1 μM FIH, 100 μM sodium ascorbate, 10 μM 2OG disodium salt, 10 μM Fe(II), 50 mM Tris–HCl (pH 7.5), and 50 mM NaCl, at ambient temperature. 2OG, 2-oxoglutarate; ASPP, apoptosis stimulating of p53 protein; FIH, factor inhibiting hypoxia-inducible factor; HA, hemagglutinin; IgG, immunoglobulin G.

Initially, we performed coimmunoprecipitation experiments involving vectors that overproduce V5-tagged WT ASPP2 (WT ASPP2-V5) and ASPP2 V5 variants with hemagglutinin (HA)-tagged WT FIH (WT HA-FIH) in human bone osteosarcoma epithelia cells (U2OS) (Fig. 1). The ASPP2-V5 variants investigated included an N986A variant without the reported (23) ASPP2 FIH hydroxylation site at Asp-986 and subsequently an ASPP2 N984A variant. Surprisingly, the alanine variants of the “VNVN” motif in ASPP2-V5 showed increased coimmunoprecipitation of HA-FIH compared with WT ASPP2-V5.

To investigate the potential extent of FIH-catalyzed ASPP ARD hydroxylation, ASPP1-V5, ASPP2-V5, and iASPP-V5 encoding vectors were transfected into U2OS and U2OS “FIH CRISPR KO” cell lines (U2OS FIH KO) that were reconstituted with WT HA-FIH, a catalytically inactive HA-FIH D201A variant or an empty vector. LC–MS/MS analysis revealed hydroxylation of iASPP-V5, ASPP1-V5, and ASPP2-V5 in an FIH-dependent manner (Figs. S1–S4). Unexpectedly, in addition to the single hydroxylation of the asparaginyl residues present in the canonical ARD hydroxylation motifs (L…NV**N**) as reported by Janke *et al.* (23), evidence for a second FIH-dependent hydroxylation site was observed at the asparagine two residues away (-2) from the canonical FIH hydroxylation site. Thus, in the cases of ASPP1-V5 and ASPP2-V5, there was evidence for hydroxylation at the V**N**VN residue (underlined), in addition to the VNV**N** residue and in the case of iASPP-V5 of the A**N**VN residue, in addition to the ANV**N** residue (Figs. S1–S4). Evidence for the double VNVN hydroxylation was only observed in samples with elevated WT HA-FIH levels. Attempts to determine the hydroxylation levels of endogenous ASPPs were unsuccessful, likely because of their relatively low abundance.

### ASPP peptides are monohydroxylated by FIH

To investigate the extent of FIH-catalyzed ASPP protein ARD hydroxylation, we then tested if ASPP peptides spanning both potential hydroxylation regions are accepted by FIH as substrates (Fig. 1). ASPP peptides (iASPP residues 670–693; ASPP1 residues 932–954; and ASPP2 residues 969–991) were incubated with FIH in the presence of appropriate cosubstrates (2OG, O_2_) and the cofactor Fe(II), under conditions reported to sustain FIH catalysis (8, 11). Following reaction, all the peptides manifested a clear +16 Da mass shift relative to the substrates, corresponding to a single hydroxylation reaction, which was shown to occur in an FIH-dependent manner (Fig. 1). The modification site was assigned for iASPP at Asn-687, using fragmentation mass spectrometry (MS) (Fig. S5 and Table S1). This (canonical) hydroxylation site corresponds to the previously assigned hydroxylation site on ASPP2 at Asn-986 (23), which is on a loop linking the second and third ARDs of ASPP2 and which is spatially adjacent to the Src homology 3 domain (Fig. S6).

**Figure 2:**
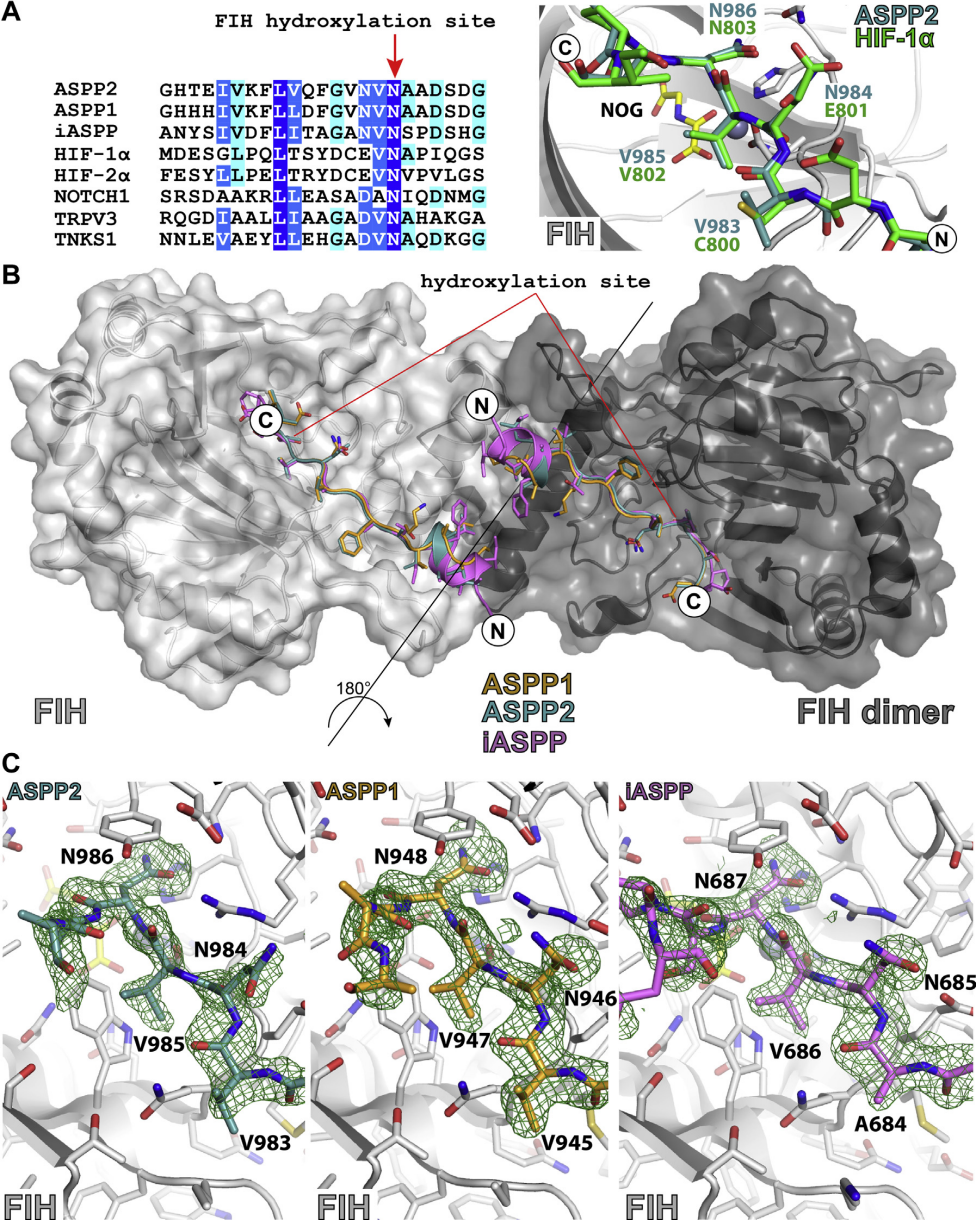
**Views from crystal structures of FIH in complex with ASPP-derived peptides.***A*, *left*, sequence alignment of ASPPs with reported FIH substrates. *Right*, overlay of crystal structure–derived views of FIH in complex with ASPP2 and HIF-1α (Protein Data Bank code: 1H2K; 2.15 Å) showing the conserved nature of substrate binding. *B*, views from the dimeric structure of FIH in complex with an iASPP-derived peptide (residues 670–693), an ASPP1-derived peptide (residues 932–954), and an ASPP2-derived peptide (residues 969–991). *C*, close-up views from crystal structures of FIH in complex with ASPP-derived peptides, the *F*o–*F*c OMIT maps, shown in *green mesh*, are contoured to 3σ. ASPP, apoptosis stimulating of p53 protein; FIH, factor inhibiting hypoxia-inducible factor; HIF, hypoxia-inducible factor.

To investigate how the ASPPs bind to FIH, we crystallized FIH in complex with iASPP-, ASPP1-, and ASPP2-derived peptides containing the VNVN/ANVN motif (Fig. 2). The structures show that, at least in the crystalline state, all the ASPP fragment peptides bind in a conserved fashion, with the “canonical” asparagine residue orientated for β-hydroxylation, including *via* hydrogen bonding with Gln-239 of FIH (16), in accord with the peptide hydroxylation data (Fig. 2). The “second” asparagine is positioned in a similar orientation compared with Glu-801 of human HIF-1α with a metal to C-β distance of >10 Å, that is, in these structures, the “second” asparagine is not productively positioned to be hydroxylated (Fig. 2). The binding of the peptides implies that for protein ASPP substrates, at least partial unfolding of the ARDs must occur in order to bind to FIH in a catalytically productive manner, as has been proposed for other ARD substrates (13). Crystallization efforts of FIH in complex with larger fragments of ASPP proteins have as yet been unsuccessful.

Overall, the biochemical and biophysical results with peptides contrast with the cellular results for ASPP1/2 and iASPP proteins, where we observed double hydroxylation, in that with the peptides, only a single hydroxylation was observed. We investigated if substrate–enzyme interactions distant from the active site (*i.e.*, not observed in the peptide substrates–FIH complexes) are needed for the VNVN double hydroxylation assigned in cells by producing recombinant forms of the C-terminal domains of iASPP, ASPP1, and ASPP2. Consistent with the cellular results, the C-terminal domains of all three proteins were subjected to FIH-catalyzed hydroxylation assays. LC–MS/MS analysis for all proteins showed evidence for double hydroxylation of the VNVN/ANVN motifs (Figs. S7–S9). The ASPPs were shown to compete with a HIF-1α fragment and a previously reported consensus ankyrin substrate (19) for FIH-catalyzed hydroxylation (Figs. S10–S12), raising the possibility that they might, along with other FIH substrates, be involved in regulating the role of FIH in the hypoxic response. Consensus ARD-derived peptides (19, 20) containing VNVN, ANVN, and VNAN motifs were also shown to be FIH substrates undergoing single hydroxylations (Fig. S13).

**Figure 3:**
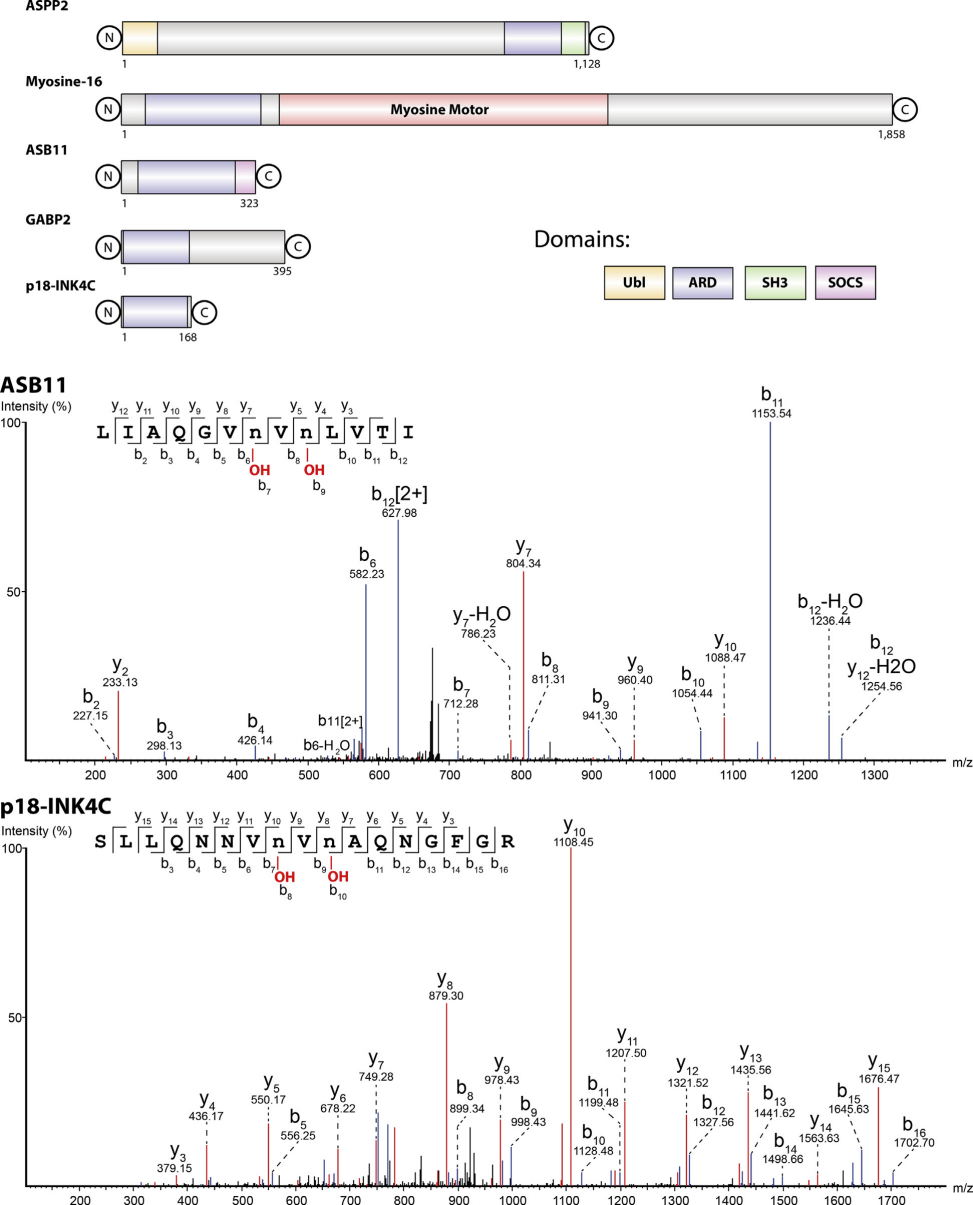
**Evidence that FIH catalyzes two hydroxylations of ASB11 and p18.***Top*, domain structure of human “VNVN” motif–containing proteins. *Middle*, LC–MS/MS spectra implying a doubly hydroxylated ASB11 peptide at Asn-92 and Asn-90 (indicated by a lowercase n). *Bottom*, LC–MS/MS spectra for doubly hydroxylated peptides at Asn-32 and Asn-30 (indicated by a lowercase n). Reaction conditions: 1 μM ASB11, 0.25 μM FIH, 1 mM sodium ascorbate, 1 mM 2OG disodium salt, 200 μM Fe(II), and Tris–HCl (pH 7.5) incubated for 3 h at 37 °C. 2OG, 2-oxoglutarate; FIH, factor inhibiting hypoxia-inducible factor.

The combined turnover and structural studies imply that in order for the second FIH-catalyzed hydroxylation to occur, interactions in addition to those at the active site (Figs 1 and S6) and which do not manifest in the FIH:peptide fragment crystal structures are involved in the second hydroxylation. To investigate if the VNVN double hydroxylation is specific to ASPPs or can be manifested with other FIH substrates, we conducted a bioinformatic search for human proteins containing a “VNVN” motif in their ankyrin repeats. In the human proteome, we found six members of the ARD family, including ASPP1 and ASPP2, that contain a VNVN motif (Fig. 3). To investigate if these manifest FIH-catalyzed single or double VNVN hydroxylations, we produced recombinant forms of ASB11 and P18-INK4C and tested them for FIH-catalyzed hydroxylation (Fig. 3). MS fragmentation studies provided evidence that ASB11 and P18-INK4C proteins undergo both monohydroxylation and dihydroxylation reactions within their VNVN motif (Figs 3, S14 and S15). Note that ASB11 has also a second assigned hydroxylation site at Asp-125 in the ARD, which is not part of a “VNVN” motif (Fig. S16). Peptides derived from ASB11 and P18-INK4C spanning potential hydroxylation regions, analogous to the ASPP peptides, were not accepted by FIH as substrates under the tested conditions (data not shown).

## Discussion

The overall results show that FIH can catalyze hydroxylation of two asparaginyl residues within the same VNVN/ANVN motifs present in ARD substrates, as shown by work with isolated recombinant proteins and by analyses of full-length proteins in cells, albeit in the latter case with elevated FIH levels (Figure 1, Figure 2, Figure 3). The ability of FIH to catalyze two hydroxylations of the same VNVN motif in the case of ASPP1/2, ASB11, P18, and ANVN, at least in the case of iASPP, within ARDs is unprecedented.

The ability of 2OG-dependent oxygenases and related oxygenases/oxidases to catalyze a range of different types of oxidative reactions and to accept multiple substrates is long established in plants and microbes (24, 25). In the case of human 2OG oxygenases acting on proteins and nucleic acids, the biologically relevant reactions catalyzed by them are presently limited to hydroxylations (including the sequential oxidation of methyl groups to acids) and N-methyl demethylations likely proceeding *via* sequential hydroxylation. However, certain 2OG-dependent protein hydroxylases, including FIH, AspH, JMJD6 and some procollagen-modifying enzymes, likely act on multiple substrates (24, 25). FIH is of particular interest because not only can it accept both HIF-α isoforms and multiple ARD (and other) proteins but also it can accept different residues as substrates, including Asn, Asp, His, and, at least in isolated form, some D-amino acids (17, 18, 26, 27) at least in the context of ARD-derived substrates.

FIH has recently been reported to form tight complexes with proteins in an oxygen-dependent manner, in particular with the deubiquitinase ovarian tumor domain containing ubiquitin aldehyde binding protein 1 (OTUB1), likely *via* covalent bond formation (28). The human 2OG-dependent prolyl hydroxylase OGFOD1 is also reported both to catalyze a single C-3 hydroxylation of its ribosomal protein 23 substrate and to form a (likely) covalently linked complex with it (29). Interestingly, the yeast homolog of human OGFOD1 is reported to catalyze both the single and apparent double hydroxylation of the analogous prolyl residue (30). In the case of substrate analog studies with isolated FIH, we have also observed that the double hydroxylation of a single residue can occur (d-Leu) though there is no evidence that this reaction is of biological relevance (27). Evidence that FIH can catalyze the desaturation of certain residues has also been accrued (17). Collectively, these observations imply that the range of oxidative reactions catalyzed by animal 2OG oxygenases, including FIH, likely extend well beyond those presently defined biochemically.

Interestingly, we observed two hydroxylations of the VNVN/ANVN motifs with protein substrates but not with ASPP-derived peptide substrates. In the case of peptide substrates, we only observed hydroxylation at the canonical asparagine site, that is, the second asparagine residue in the VNVN or ANVN motifs. These observations are in accord with proposals that enzyme–substrate interactions relatively distant from the FIH active site, likely involving at least partial unfolding of the stereotypically observed ARD conformation, are involved in regulating catalysis by FIH (13, 31). The observation of somewhat different conformations of the VNVN-containing regions of ASPP1 and ASPP2 when complexed with FIH (Fig. 2) compared with their crystal structures when not complexed with FIH (32, 33) is consistent with this proposal. Thus, we propose that specific and likely dynamic interactions between FIH and VNVN/ANVN-containing ARD proteins enable the “second” noncanonical FIH substrate asparagine residue (V**N**VN/A**N**VN) to be positioned productively for hydroxylation. The details of these conformational changes, along with the cellular biological roles of the double hydroxylation, are the subject of current investigations.

## Experimental procedures

Recombinant forms of FIH (full length), ASPP2_889-__1128_-Avi-His_6_, and iASPP_625-__828_-Avi-His_6_, were produced and purified as described (8, 34). Peptides (all with C-terminal amides) were from GL Biochem. l-(+)-ascorbic acid sodium salt (code: 11140), ammonium iron (II) sulfate hexahydrate (code: 215406), *N*-oxalylglycine, and 2OG disodium salt hydrate (K3752) were from Sigma–Aldrich.

### Recombinant protein purification

Recombinant proteins were produced by standard procedures. DNA encoding for ASPP1_883–1090_ was cloned into the pGEX-6P2 vector between BamHI and NotI restriction sites. The ASPP1_883–1090_ encoding construct was transformed into *Escherichia coli* BL21(DE3) cells and grown at 37 °C in LB–Miller medium containing 50 μg/ml ampicillin. Recombinant protein production was induced by addition of IPTG (final concentration of 0.5 mM) for 16 h at 18 °C. Cells were lysed by sonication, centrifuged (34,000*g*, 30 min), and filtered (0.45 μm filter). The glutathione-*S*-transferase (GST)-tagged protein was purified using a Glutathione Sepharose column (GE Healthcare). Fractions containing the desired protein were combined, buffer exchanged, and the GST tag was cleaved using the viral 3C protease. The cleaved GST tag was removed using a Glutathione Sepharose column. The resultant cleaved protein was further purified by size-exclusion chromatography (Superdex-75 column; GE Healthcare).

The recombinant P18-INK4C production vector was a kind gift from Prof Jane Endicott; P18-INK4C protein production was induced by the addition of IPTG (final concentration of 0.5 mM) for 16 h at 18 °C, prior to cell harvesting. P18-INK4C containing cell pellets were resuspended in 50 mM Hepes–NaOH, 150 mM NaCl, 2 mM DTT, pH 7.5 buffer, lysed by sonication, and cleared of cell debris by centrifugation and filtration prior to immobilizing the GST-tagged proteins on a Glutathione Sepharose column. The column was washed with the same buffer (100 ml) prior to elution with 40 mM Hepes–NaOH, 200 mM NaCl, 10 mM reduced glutathione, and pH 7.5 buffer. Fractions containing the desired protein were pooled, the viral 3C protease (1:50 ratio) added, and the mixture was incubated overnight at 4 °C. The GST cleaved protein was further purified by size-exclusion chromatography using a Superdex-75 column and 40 mM Hepes–NaOH, 200 mM NaCl, and pH 7.4 as the running buffer. DNA encoding for ASB11_64–287_ was a kind gift from Dr Alex Bullock; the protein was purified as described: https://www.thesgc.org/structures/4UUC.

### X-ray crystallography

Crystals of FIH substrate complexes were grown in sitting drops (300 nl) using the vapor diffusion method at 293 K in a protein to reservoir ratio of 2:1. The FIH solution contained 11 mg/ml FIH (0.27 mM) in 50 mM Tris–HCl (pH 7.5), *N*-oxalylglycine (1 mM), and the appropriate substrate peptide (2 mM) (16). For crystallization purposes, Fe(II) was substituted for Zn(II) to avoid metal oxidation. Crystals were cryoprotected by transfer into the crystallization buffer supplemented with 20% (v/v) glycerol and freeze cooled by plunging into liquid nitrogen. Data were collected from single crystals at 100 K using Diamond MX beamlines (Tables S2 and S3). The structures were solved using Phaser (35) using Protein Data Bank code 1H2K as a search model. Alternating cycles of refinement using PHENIX (36) and model building using COOT (37) were performed until *R*_work_ and *R*_free_ converged.

### LC–MS peptide hydroxylation assays

All kinetic measurements were performed by monitoring the appearance of hydroxylated substrate peptides using a RapidFire RF365 high-throughput sampling robot (Agilent) connected to an Agilent 6550 accurate mass quadrupole time-of-flight mass spectrometer equipped with an Agilent jet stream electrospray ionization source (38). l-ascorbic acid (50 mM in deionized water), 2OG (10 mM in deionized water), and iron (II) sulfate (400 mM in 10 mM HCl) were prepared freshly each day. For each assay, 50 mM Tris–HCl (pH 7.5), 50 mM NaCl (prepared freshly each day) containing 100 μM l-ascorbic acid, 10 μM ammonium (II) sulfate, 10 μM 2OG, and 10 μM peptide substrate were added to a well of a 96-deep well polypropylene plate (Greiner Bio-One). The plate was then transferred to a RapidFire (RF365) high-throughput sampling robot, and reaction was initiated by adding FIH to a final concentration of 0.15 μM. Enzyme reactions were performed at room temperature, and assay samples were aspirated from the plate and loaded onto a C4 solid phase extraction (SPE) cartridge. The C4 SPE was then washed with LC–MS grade water containing 0.1% (v/v) aqueous formic acid for 5 s to remove nonvolatile buffer salts, and then the peptide was eluted from the C4 SPE with an organic solvent for 5 s consisting of 85% (v/v) LC–MS grade acetonitrile, 15% (v/v) LC–MS grade water, and 0.1% (v/v) formic acid. The whole cycle of sample loading, aqueous wash, and organic solvent elution takes approximately 12 s, thus enabling accurate kinetic measurements by MS. Peptide charge states were monitored in the positive electrospray ionization mode with a drying gas temperature of 280 °C, a drying gas flow rate of 13 l/min, nebulizer gas pressure of 40 psi, sheath gas temperature of 350 °C, sheath gas flow rate of 12 l/min, and a nozzle voltage of 1000 V. Ion chromatogram data were isolated for both the peptide substrate and the hydroxylated peptide substrate and integrated using RapidFire integrator software (Agilent). The percent conversion of the peptide substrate to the +16 hydroxylated peptide was calculated using the equation: % conversion = 100 × hydroxylated/(hydroxylated + nonhydroxylated peptide).

### MALDI–MS hydroxylation assays

MALDI–MS measurements were performed using a Bruker Ultraflex instrument as reported (39). l-ascorbic acid (10 mM in deionized water), 2OG (10 mM in deionized water), and iron (II) sulfate (2 mM in 10 mM HCl) were prepared freshly each day. For each assay, FIH (4 μM) was added to 50 mM Tris–HCl (pH 7.5), containing 1 mM l-ascorbic acid, 200 μM ammonium iron (II) sulfate, 1 mM 2OG, and 100 μM peptide substrate and incubated at 37 °C. Reactions were halted by spotting samples onto a target plate for MALDI–MS, mixing with either α-cyano-4-hydroxycinnamic or sinapinic acid (10 mg/ml in 50% [v/v] acetonitrile and 0.1% [v/v] formic acid). Hydroxylation levels were determined as reported (40). MALDI–MS/MS measurements were performed as reported (15).

### Protein hydroxylation assays

l-ascorbic acid (10 mM in deionized water), 2OG (10 mM in deionized water), and iron (II) sulfate (2 mM in 10 mM HCl) were prepared freshly each day. For each assay, FIH (0.5–1 μM) was added to 50 mM Tris–HCl (pH 7.5), containing 1 mM l-ascorbic acid, 200 μM ammonium iron (II) sulfate, 1 mM 2OG, and 1 to 2 μM protein substrate and incubated at 37 °C. Reactions were quenched by methanol/chloroform precipitation and proteins were digested using elastase according to as reported (41).

### Cell culture and immunoprecipitation

Details of the FIH KO cell line will be reported elsewhere. U2OS and U2OS FIH KO cells were cultured in Dulbecco's modified Eagle's medium (Gibco Life Technologies) supplemented with 10% (v/v) fetal bovine serum (Invitrogen), 4 mM l-glutamine, 100 U/ml penicillin, and 100 μg/ml streptomycin (Gibco Life Technologies) at 37 °C and 5% CO_2_. Cells were seeded to reach 60 to 80% confluency prior to transfection using FuGENE 6 transfection reagent (Promega) according to the manufacturer’s protocol. Cell monolayers were washed with PBS and lysed for 30 min in EDTA, 20 mM Tris–HCl (pH 7.4), 0.5% Nonidet P-40, and 150 mM MgCl_2_ supplemented with protease and phosphatase inhibitors. Cell lysates were incubated for 3 h with the anti-V5 antibody (MCA1360; Serotec) prior to addition of Dynabeads protein G beads (Thermo Fisher Scientific) for 60 min. Immunoprecipitated samples were washed three times with 150 mM NaCl, 5 mM EDTA, 50 mM Tris–HCl (pH 7.5), and 0.5% Nonidet P-40 buffer and eluted from the beads by boiling (5 min) in 2× concentrated Laemmli sample buffer (Sigma–Aldrich). Isolated proteins were separated *via* SDS-PAGE on gradient SDS-PAGE gels according to the manufacturers’ protocol (Life Technologies).

### LC–MS/MS analyses

Proteins of interest were transfected as described previously; immunoprecipitated proteins were separated on gradient SDS-PAGE gels (Life Technologies), and bands were visualized using Instant Blue stain (Expedeon). Protein bands of interest were excised from the gel and then digested using elastase (Worthington Biochemicals) and analyzed using PEAKS7 with a 1% false discovery rate.

## Data availability

All data are contained within the article. The MS proteomics data including the sample processing protocol and search parameters have been deposited to the ProteomeXchange Consortium *via* the PRIDE (42) partner repository with the dataset identifier PXD017278 (Project DOI: 10.6019/PXD017278).

## Supporting information

This article contains supporting information.

## Conflict of interest

The authors declare that they have no conflicts of interest with the contents of this article.
