# Supplementary material for: Factor inhibiting HIF can catalyze two asparaginyl hydroxylations in VNVN motifs of ankyrin fold proteins
Source: J Biol Chem. 2022 May 7;298(6):102020. doi: 10.1016/j.jbc.2022.102020 (PMC9189129; doi:10.1016/j.jbc.2022.102020)
Supplement: Supplemental information [file mmc1.pdf]

Supplemental Information

Factor Inhibiting HIF catalyses asparaginyl hydroxylations in VNVN motifs in some ankyrin proteins

Thomas M. Leissing[1,2], Adam P. Hardy[1], Hokfung Chan[2], Yihua Wang[2], Anthony Tumber[1], Rasheduzzaman Chowdhury[1], Penny Fang[4], Mathew L. Coleman[5], Matthew E. Cockman[3], Holger B. Kramer[6,7], Georgina Berridge[4], Roman Fischer[4], Benedikt M. Kessler[4], Peter J. Ratcliffe[2, 3], Xin Lu[2] and Christopher J. Schofield[1]

Addresses: [1]Chemistry Research Laboratory, Department of Chemistry and the Ineos Oxford Institute for Antimicrobial Research, Mansfield Road, University of Oxford, Oxford, OX1 3TA, U.K.; [2]Ludwig Institute for Cancer Research, Nuffield Department of Clinical Medicine, University of Oxford, U.K.; [3]The Francis Crick Institute, London, U.K. [4]NDM Research Building, University of Oxford, Oxford, U.K.; [5]Institute of Cancer and Genomic Sciences, University of Birmingham, Edgbaston, Birmingham B15 2TT, U.K., [6]MRC London Institute of Medical Sciences, Du Cane Road, London W12 0NN, U.K.; [7]Institute of Clinical Sciences, Imperial College London, Hammersmith Hospital Campus, Du Cane Road, London W12 0NN, U.K.

Supplementary Figures

**Figure S1 Overview of hydroxylation assays of V5-tagged ASPP proteins and FIH variants in U2OS cells.**	**3**
**Figure S2 FIH mediated hydroxylation of full-length V5-iASPP in U2OS cells.**	**5**
**Figure S3 FIH mediated hydroxylation of full-length ASPP1-V5 in U2OS cells.**	**7**
**Figure S4 FIH mediated hydroxylation of full-length ASPP2-V5 in U2OS cells.**	**9**
**Figure S5 FIH hydroxylates an iASPP derived peptide (residues 670-693).**	**10**
**Figure S6 Sequence alignments of human ankyrin repeats containing a “VNVN” tetrapeptide sequence motif.**	**11**
**Figure S7 FIH catalysed hydroxylation of recombinant iASPP (625-828) protein.**	**13**
**Figure S8 FIH catalysed hydroxylation of recombinant ASPP1 (883-1090) protein.**	**15**
**Figure S9 FIH catalysed hydroxylation of recombinant ASPP2 (889-1128) protein.**	**17**
**Figure S10 ASPP proteins compete with HIF-α for FIH catalysed reactions.**	**18**
**Figure S11 Competition of ARD proteins with HIF-1α for recombinant isolated FIH activity.**	**19**
**Figure S12 Half maximal inhibitory concentration (IC_{50}) measurements of ASPP1 (883-1090) and mNOTCH1 (1899-2106) competition for FIH mediated HIF-1α (789-822) hydroxylation.**	**20**
**Figure S13 FIH hydroxylation assays of consensus ankyrin repeat domain (1CA) derived peptides bearing a “VNVN”, “ANVN” or “VNAN” motif.**	**21**
**Figure S14 FIH catalysed hydroxylation of recombinant full-length p18-INK4C protein.**	**23**
**Figure S15 FIH catalysed hydroxylation of recombinant ASB11 (64-287) protein.**	**25**
**Figure S16 FIH catalysed hydroxylation of recombinant ASB11 (64-287) protein at Asn-125.**	**26**

Supplementary Tables

**Table S1 Ion tables for MS-MS spectra from MALDI LIFT-TOF-TOF analysis of the FIH hydroxylation site of the iASPP (670-693) peptide (see Figure S5).**	**27**
**Table S2 Data collection and refinement statistics of FIH complexes with ASPP peptides substrates**	**28**
**Table S3 Buffer and vapour diffusion conditions used for FIH complex crystallisation.**	**29**

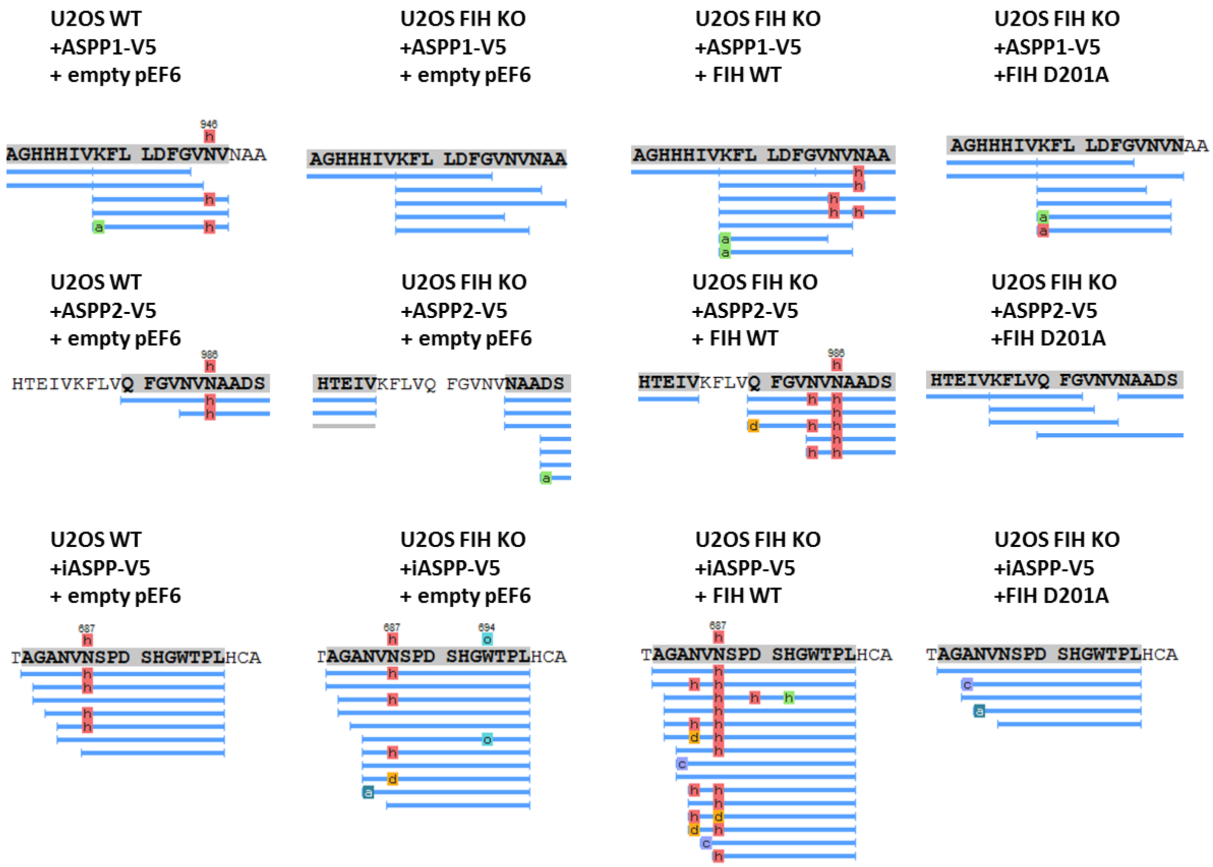

Figure S1 Overview of hydroxylation assays of V5-tagged ASPP proteins and FIH variants in U2OS cells. Recombinant V5-tagged full-length ASPP1 / ASPP2 / iASPP were produced in U2OS cells or U2OS FIH KO cells bearing an empty pEF6 vector or vectors encoding for production of FIH WT or FIH D201A. Proteins were immunoprecipitated with an anti-V5 antibody and digested with elastase. Blue lines indicate identified peptides of the digested protein. A red background indicates an annotated hydroxylation site. Data were analysed using PEAKS7 software (Bioinformatics Solutions).

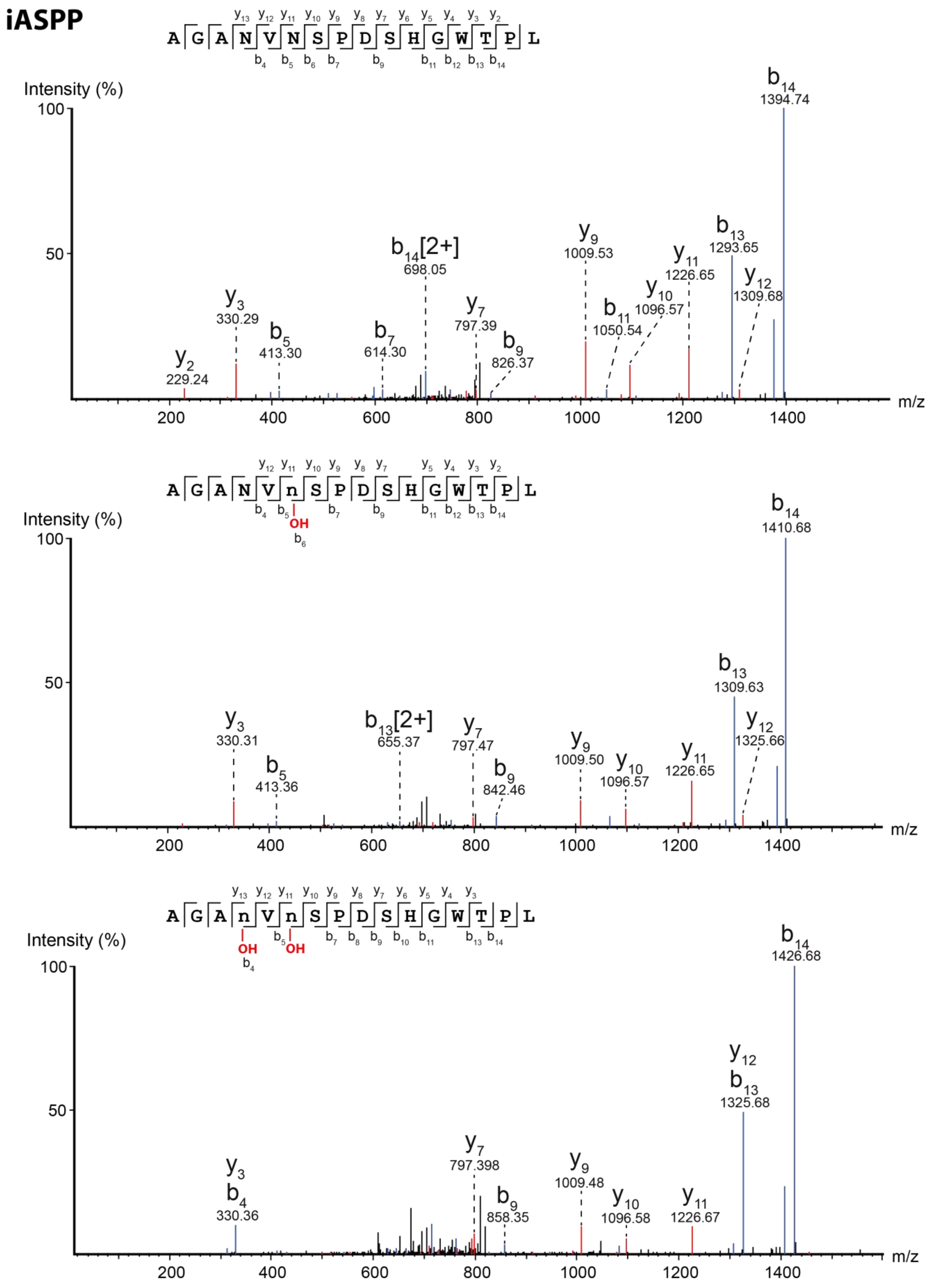

(Figure continues on next page)

iASPP

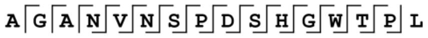

#	b	b-H2O	b-NH3	b (2+)	Seq	y	y-H2O	y-NH3	y (2+)	#
1	72.04	54.03	55.02	36.52	A					16
2	129.07	111.06	112.04	65.03	G	1551.72	1533.71	1534.69	776.42	15
3	200.10	182.09	183.08	100.55	A	1494.70	1476.69	1477.67	747.87	14
4	314.37	296.25	297.34	157.57	N	1423.81	1405.65	1406.63	712.50	13
5	413.30	395.29	396.27	207.11	V	1309.68	1291.61	1292.32	655.31	12
6	527.35	509.29	510.25	264.13	N	1210.65	1192.64	1193.52	605.77	11
7	614.30	596.35	597.31	307.64	S	1096.60	1078.57	1079.48	548.75	10
8	711.34	693.33	694.49	356.17	P	1009.53	991.57	992.45	505.24	9
9	826.37	808.36	809.34	413.68	D	912.51	894.41	895.32	456.71	8
10	913.40	895.32	896.63	457.20	S	797.39	779.38	780.37	399.20	7
11	1050.54	1032.68	1033.55	525.73	H	710.48	692.35	693.34	355.68	6
12	1107.54	1089.47	1090.45	554.24	G	573.27	555.26	556.42	287.15	5
13	1293.65	1275.61	1276.64	647.54	W	516.45	498.33	499.25	258.64	4
14	1394.74	1376.72	1377.58	698.05	T	330.29	312.32	313.18	165.60	3
15	1491.66	1473.65	1474.63	746.58	P	229.24	211.14	212.13	115.08	2
16					L	132.10	114.09	115.07	66.55	1

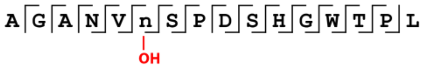

#	b	b-H2O	b-NH3	b (2+)	Seq	y	y-H2O	y-NH3	y (2+)	#
1	72.04	54.03	55.02	36.52	A					16
2	129.07	111.06	112.04	65.03	G	1567.71	1549.70	1550.69	784.25	15
3	200.10	182.09	183.08	100.55	A	1510.69	1492.68	1493.67	755.83	14
4	314.24	296.14	297.12	157.57	N	1439.65	1421.73	1422.63	720.29	13
5	413.36	395.23	396.26	207.11	V	1325.66	1307.82	1308.59	663.31	12
6	543.15	525.24	526.17	272.13	N(+15.99)	1226.65	1208.64	1209.52	613.77	11
7	630.27	612.27	613.26	315.64	S	1096.57	1078.50	1079.48	548.75	10
8	727.34	709.33	710.31	364.17	P	1009.50	991.53	992.45	505.17	9
9	842.46	824.35	825.52	421.68	D	912.56	894.23	895.54	456.71	8
10	929.40	911.39	912.56	465.47	S	797.47	779.54	780.37	399.20	7
11	1066.48	1048.44	1049.43	533.73	H	710.36	692.35	693.36	355.68	6
12	1123.58	1105.47	1106.55	562.24	G	573.40	555.29	556.45	287.15	5
13	1309.63	1291.52	1292.71	655.37	W	516.49	498.42	499.25	258.64	4
14	1410.68	1392.69	1393.58	705.80	T	330.31	312.24	313.18	165.60	3
15	1507.66	1489.65	1490.63	754.52	P	229.21	211.14	212.13	115.08	2
16					L	132.10	114.09	115.07	66.55	1

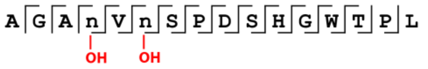

#	b	b-H2O	b-NH3	b (2+)	Seq	y	y-H2O	y-NH3	y (2+)	#
1	72.04	54.03	55.02	36.52	A					16
2	129.07	111.06	112.04	65.03	G	1583.71	1565.70	1566.68	792.31	15
3	200.10	182.09	183.08	100.55	A	1526.69	1508.68	1509.82	763.86	14
4	330.36	312.33	313.11	165.57	N(+15.99)	1455.70	1437.64	1438.62	728.49	13
5	429.32	411.37	412.18	215.10	V	1325.68	1307.69	1308.59	663.41	12
6	559.25	541.36	542.38	280.12	N(+15.99)	1226.67	1208.75	1209.58	613.77	11
7	646.38	628.36	629.25	323.64	S	1096.58	1078.61	1079.48	548.75	10
8	743.31	725.32	726.41	372.17	P	1009.48	991.47	992.29	505.34	9
9	858.35	840.52	841.33	429.68	D	912.22	894.41	895.39	456.71	8
10	945.47	927.35	928.53	473.20	S	797.39	779.38	780.37	399.18	7
11	1082.52	1064.44	1065.57	541.73	H	710.31	692.38	693.57	355.68	6
12	1139.47	1121.38	1122.44	570.30	G	573.21	555.28	556.28	287.15	5
13	1325.68	1307.69	1308.52	663.41	W	516.53	498.32	499.30	258.64	4
14	1426.68	1408.66	1409.57	713.97	T	330.36	312.33	313.18	165.60	3
15	1523.65	1505.64	1506.62	762.57	P	229.15	211.14	212.13	115.08	2
16					L	132.10	114.09	115.07	66.55	1

Figure S2 FIH mediated hydroxylation of full-length V5-iASPP in U2OS cells. LC-MS/MS analysis of iASPP-V5 in U2OS FIH CRIPSR KO cells co-transfected with pcDNA3 (ThermoFisher) vectors encoding for iASPP-V5 and FIH-HA. The spectrum shows fragments from the elastase catalysed digestion of iASPP providing evidence for hydroxylation at Asp-685 and Asp-687. Conditions: The vectors encoding for iASPP-V5 and FIH-HA were transfected, then overexpressed for 24 h in U2OS FIH CRISPR KO cells. Immunoprecipitation employed an anti-V5 antibody. Proteins were separated by SDS-PAGE; the band corresponding to iASPP-V5 was excised and the protein digested using elastase (16 h, 37 °C).

ASPP1

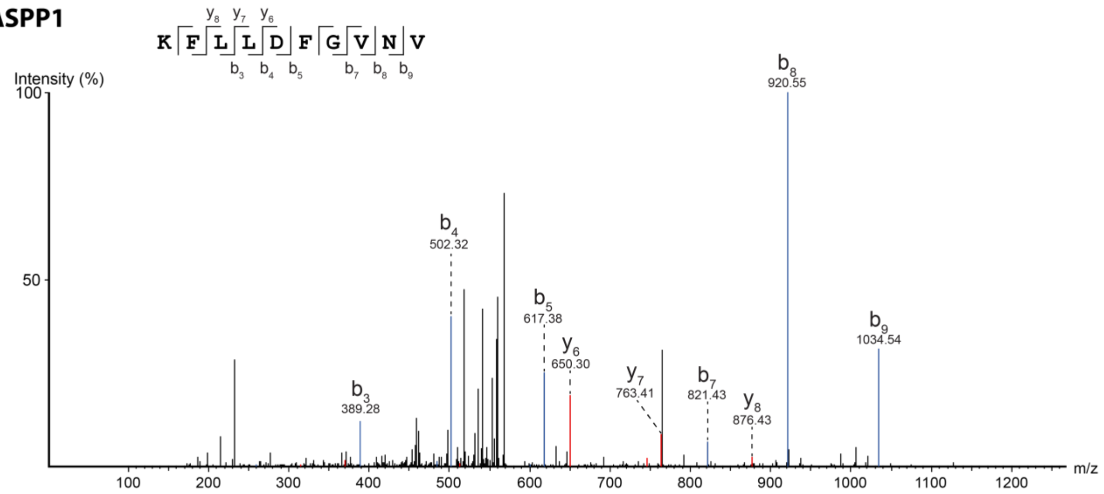

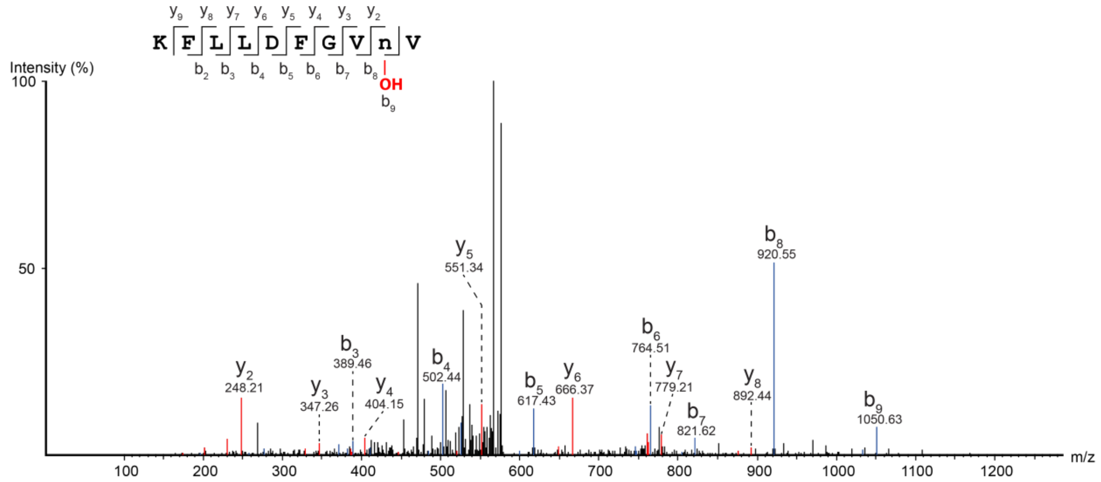

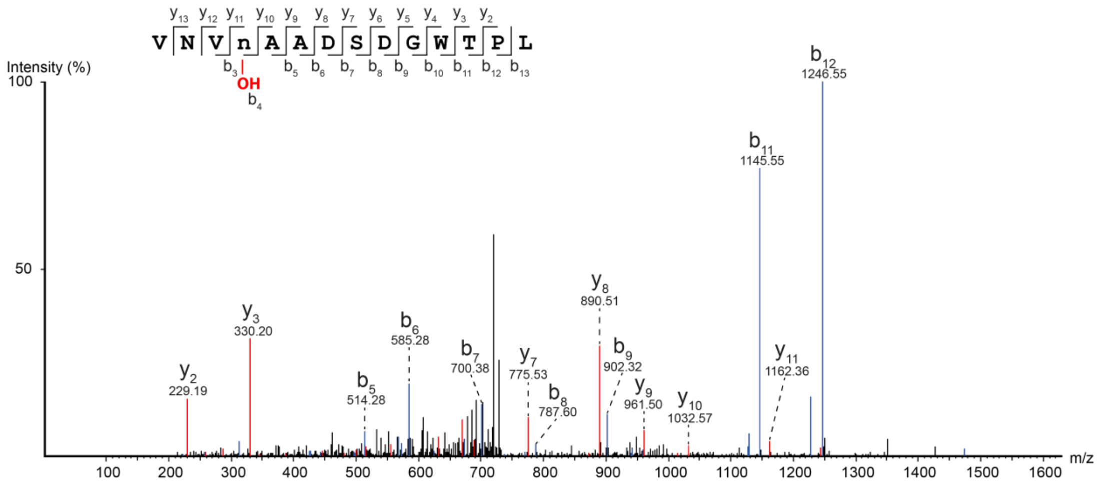

(Figure continues on next page)

ASPP1

K F L L D F G V N V

#	b	b-H2O	b-NH3	b (2+)	Seq	y	y-H2O	y-NH3	y (2+)	#
1	129.10	111.09	112.08	65.05	K					10
2	276.17	258.16	259.19	138.59	F	1023.55	1005.54	1006.52	512.24	9
3	389.28	371.24	372.23	195.13	L	876.43	858.47	859.46	438.74	8
4	502.32	484.38	485.31	251.67	L	763.41	745.39	746.37	382.20	7
5	617.38	599.36	600.33	309.18	D	650.30	632.30	633.29	325.66	6
6	764.43	746.42	747.41	382.72	F	535.29	517.28	518.26	268.14	5
7	821.43	803.45	804.40	411.23	G	388.22	370.24	371.19	194.61	4
8	920.55	902.51	903.50	460.76	V	331.20	313.19	314.20	166.10	3
9	1034.54	1016.59	1017.54	517.78	N	232.13	214.12	215.10	116.56	2
10					V	118.09	100.08	101.06	59.54	1

K F L L D F G V n V
OH

#	b	b-H2O	b-NH3	b (2+)	Seq	y	y-H2O	y-NH3	y (2+)	#
1	129.10	111.09	112.08	65.05	K					10
2	276.34	258.29	259.14	138.59	F	1039.55	1021.54	1022.52	520.72	9
3	389.46	371.34	372.23	195.59	L	892.44	874.43	875.38	446.83	8
4	502.44	484.41	485.31	251.67	L	779.21	761.70	762.37	390.20	7
5	617.43	599.20	600.20	309.18	D	666.37	648.78	649.28	333.65	6
6	764.51	746.48	747.78	382.26	F	551.34	533.27	534.26	276.34	5
7	821.62	803.45	804.57	411.21	G	404.15	386.17	387.48	202.30	4
8	920.55	902.51	903.61	460.76	V	347.26	329.25	330.17	174.34	3
9	1050.63	1032.54	1033.32	525.71	N(+15.99)	248.21	230.19	231.10	124.56	2
10					V	118.09	100.08	101.06	59.54	1

V N V n A A D S D G W T P L
OH

#	b	b-H2O	b-NH3	b (2+)	Seq	y	y-H2O	y-NH3	y (2+)	#
1	100.08	82.07	83.05	50.54	V					14
2	214.12	196.11	197.09	107.56	N	1375.61	1357.78	1358.59	688.13	13
3	313.27	295.18	296.16	157.09	V	1261.57	1243.56	1244.54	631.34	12
4	443.32	425.23	426.34	222.42	N(+15.99)	1162.36	1144.49	1145.55	581.75	11
5	514.28	496.28	497.56	257.36	A	1032.57	1013.99	1015.44	516.73	10
6	585.28	567.50	568.37	293.15	A	961.50	943.42	944.24	481.34	9
7	700.38	682.32	683.30	350.66	D	890.51	872.38	872.91	445.41	8
8	787.60	769.20	770.35	394.18	S	775.53	757.35	758.34	387.91	7
9	902.32	884.38	885.36	451.69	D	688.13	670.17	671.30	345.15	6
10	959.23	940.98	942.26	480.20	G	573.41	555.23	556.24	287.24	5
11	1145.55	1127.60	1128.49	573.41	W	516.21	498.32	499.27	258.27	4
12	1246.55	1228.57	1229.51	624.13	T	330.20	312.48	313.27	165.60	3
13	1343.59	1325.58	1326.56	672.69	P	229.19	211.14	212.13	115.08	2
14					L	132.10	114.09	115.07	66.55	1

Figure S3 FIH mediated hydroxylation of full-length ASPP1-V5 in U2OS cells. LC-MS/MS analysis of ASPP1-V5 in U2OS FIH CRIPSR KO cells co-transfected with pcDNA3 (ThermoFisher) vectors encoding for ASPP1-V5 and FIH-HA. The spectrum shows fragments from the elastase catalysed digestion of ASPP1 Asp-946 and Asp-948. Conditions: The vectors encoding for ASPP1-V5 and FIH-HA were transfected, then overexpressed for 24 h in U2OS FIH CRISPR KO cells. Immunoprecipitation employed an anti-V5 antibody. Proteins were separated by SDS-PAGE**;** the band corresponding to ASPP1-V5 was excised and the protein digested using elastase (16 h, 37 °C).

ASPP2

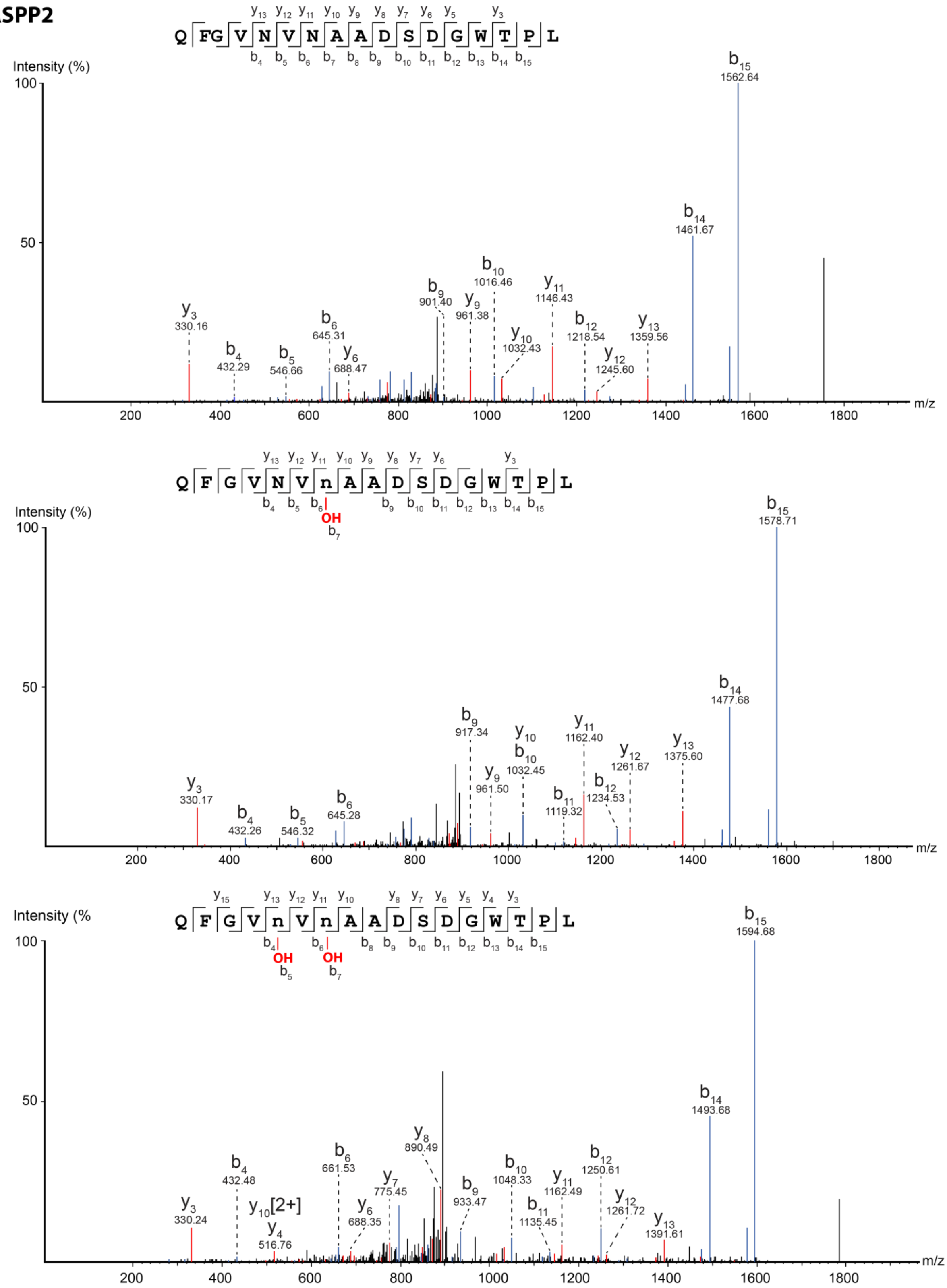

(Figure continues on next page)

ASPP2

Q FG V N V N A A D S D G W T P L

#	b	b-H2O	b-NH3	b (2+)	Seq	y	y-H2O	y-NH3	y (2+)	#
1	129.07	111.06	112.04	65.03	Q					17
2	276.13	258.12	259.11	138.57	F	1662.78	1644.77	1645.75	832.19	16
3	333.16	315.29	316.13	167.08	G	1515.71	1497.70	1498.68	758.35	15
4	432.29	414.31	415.27	216.61	V	1458.69	1440.72	1441.66	729.73	14
5	546.66	528.26	529.15	273.63	N	1359.56	1341.72	1342.59	680.31	13
6	645.31	627.25	628.31	323.17	V	1245.60	1227.59	1228.80	623.33	12
7	759.48	741.37	742.41	380.19	N	1146.43	1128.45	1129.48	573.27	11
8	830.29	812.41	813.09	415.27	A	1032.43	1014.45	1015.44	516.73	10
9	901.40	883.01	884.41	451.41	A	961.38	943.30	944.40	481.21	9
10	1016.46	998.47	999.45	508.74	D	890.44	872.39	873.13	445.69	8
11	1103.51	1085.53	1086.49	551.76	S	775.29	757.17	758.34	388.18	7
12	1218.54	1200.53	1201.03	609.77	D	688.47	670.32	671.12	344.67	6
13	1275.50	1257.55	1258.53	638.28	G	573.27	555.19	556.28	287.15	5
14	1461.67	1443.71	1444.61	731.59	W	516.28	498.06	498.90	258.64	4
15	1562.64	1544.63	1545.66	781.98	T	330.16	312.19	313.18	165.60	3
16	1659.74	1641.73	1642.71	830.29	P	229.15	211.14	212.13	115.08	2
17					L	132.10	114.09	115.07	66.55	1

Q F G V N V n A A D S D G W T P L

OH

#	b	b-H2O	b-NH3	b (2+)	Seq	y	y-H2O	y-NH3	y (2+)	#
1	129.07	111.06	112.04	65.03	Q					17
2	276.13	258.12	259.11	138.57	F	1678.77	1660.76	1661.74	839.63	16
3	333.16	315.15	316.13	167.08	G	1531.70	1513.69	1514.68	766.28	15
4	432.26	414.22	415.56	216.61	V	1474.68	1456.59	1457.65	737.98	14
5	546.32	528.60	529.31	273.63	N	1375.60	1357.68	1358.59	688.18	13
6	645.28	627.23	628.29	323.17	V	1261.67	1243.56	1244.58	631.28	12
7	775.31	757.46	758.35	388.19	N(+15.99)	1162.40	1144.50	1145.61	581.75	11
8	846.41	828.40	829.56	423.71	A	1032.45	1014.58	1015.44	516.73	10
9	917.34	899.44	900.42	459.22	A	961.50	943.42	944.65	481.21	9
10	1032.45	1014.58	1015.45	516.74	D	890.31	872.01	873.73	445.69	8
11	1119.32	1101.50	1102.36	560.25	S	775.31	757.46	758.34	388.18	7
12	1234.53	1216.52	1217.70	617.31	D	688.18	670.38	671.30	345.11	6
13	1291.56	1273.54	1274.53	646.28	G	573.30	555.29	556.28	287.15	5
14	1477.68	1459.78	1460.72	739.60	W	516.28	498.27	499.25	258.64	4
15	1578.71	1560.67	1561.66	790.08	T	330.17	312.19	313.18	165.60	3
16	1675.74	1657.72	1658.71	838.18	P	229.15	211.14	212.13	115.08	2
17					L	132.10	114.09	115.07	66.55	1

Q F G V n V n A A D S D G W T P L

OH OH

#	b	b-H2O	b-NH3	b (2+)	Seq	y	y-H2O	y-NH3	y (2+)	#
1	129.07	111.06	112.04	65.03	Q					17
2	276.13	258.12	259.11	138.57	F	1694.77	1676.76	1677.74	848.28	16
3	333.16	315.36	316.13	167.08	G	1547.72	1529.69	1530.67	774.35	15
4	432.48	414.21	415.22	216.61	V	1490.68	1472.48	1473.85	746.11	14
5	562.26	544.25	545.24	281.13	N(+15.99)	1391.61	1373.69	1374.58	696.20	13
6	661.53	643.46	644.30	331.17	V	1261.72	1243.38	1244.54	631.07	12
7	791.43	773.36	774.34	396.18	N(+15.99)	1162.49	1144.65	1145.47	581.88	11
8	862.28	844.40	845.38	431.70	A	1032.40	1014.45	1015.09	516.76	10
9	933.47	915.34	916.06	467.22	A	961.43	943.42	944.40	481.21	9
10	1048.33	1030.55	1031.44	525.13	D	890.49	872.72	873.36	445.69	8
11	1135.45	1117.46	1118.47	568.25	S	775.45	757.42	758.34	388.18	7
12	1250.61	1232.81	1233.50	626.25	D	688.35	670.32	671.31	344.67	6
13	1307.68	1289.54	1290.52	654.28	G	573.34	555.29	556.41	287.15	5
14	1493.68	1475.89	1476.60	747.31	W	516.76	498.58	499.25	258.64	4
15	1594.68	1576.76	1577.65	797.63	T	330.24	312.19	313.18	165.60	3
16	1691.73	1673.72	1674.70	846.31	P	229.15	211.14	212.13	115.08	2
17					L	132.10	114.09	115.07	66.55	1

Figure S4 FIH mediated hydroxylation of full-length ASPP2-V5 in U2OS cells. LC-MS/MS analysis of ASPP2-V5 in U2OS FIH CRIPSR KO cells co-transfected with pcDNA3 (ThermoFisher) vectors encoding for ASPP1-V5 and FIH-HA. The spectrum shows fragments from the elastase catalysed digestion of ASPP2 providing evidence for hydroxylation at Asp-984 and Asp-986. Conditions: ASPP1-V5 in pcDNA3. The vectors encoding for ASPP2-V5 and FIH-HA were transfected, then overexpressed for 24 h in U2OS FIH CRISPR KO cells. Immunoprecipitation employed an anti-V5 antibody. Proteins were separated by SDS-PAGE; the band corresponding to ASPP2-V5 was excised and the protein digested using elastase (16 h, 37 °C).

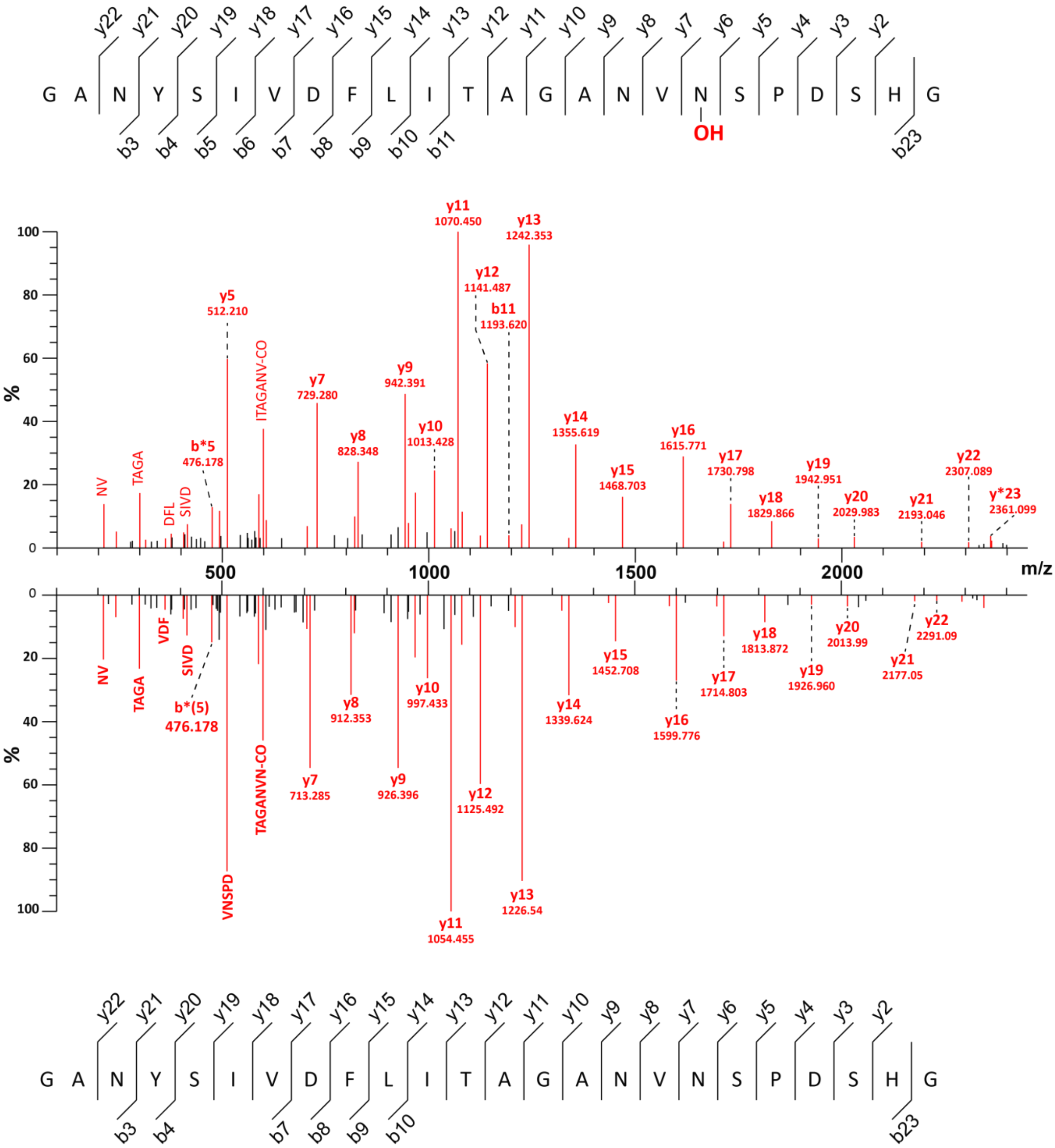

Figure S5 FIH hydroxylates an iASPP derived peptide (residues 670-693). MS-MS spectra from MALDI LIFT-TOF-TOF analysis of the FIH catalysed hydroxylation iASPP (670-639) shows evidence for hydroxylation of Asn-687. Conditions: 50 µM peptide, 4 µM FIH, 1 mM ascorbate, 1 mM 2OG, 200 µM Fe(II), 50 mM Tris-HCl pH 7.5, incubation for 30 min at 37 °C.

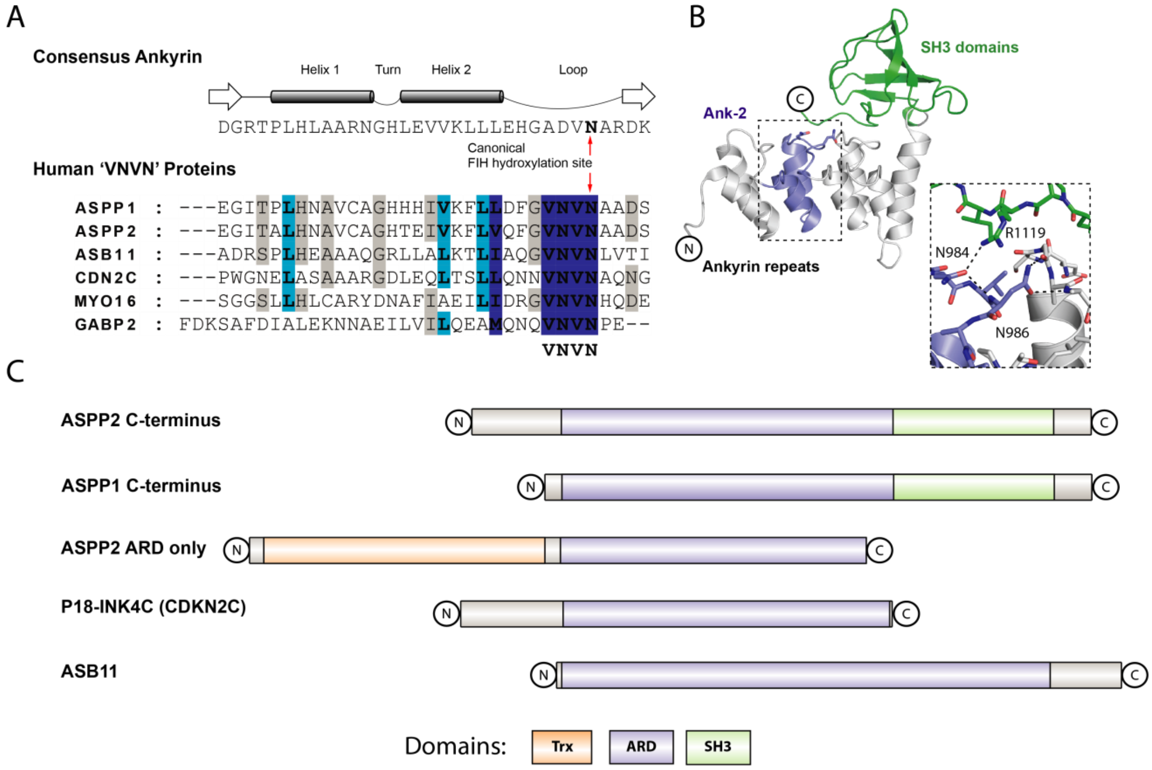

Figure S6 Sequence alignments of human ankyrin repeats containing a "VNVN" tetrapeptide sequence motif. A Sequence alignment of human "VNVN" ankyrin repeats illustrates the position of the putative hydroxylation sites within the ankyrin domain sequence. **B** View from a crystal structure of the C-terminus of ASPP2 (2) (generated from PDB 4A63, 2.27 Å) illustrating inter-domain and intra-domain interactions of the hydroxylated residues within the ASPP2 protein. **C** Domain architecture of protein constructs used for protein hydroxylation studies.

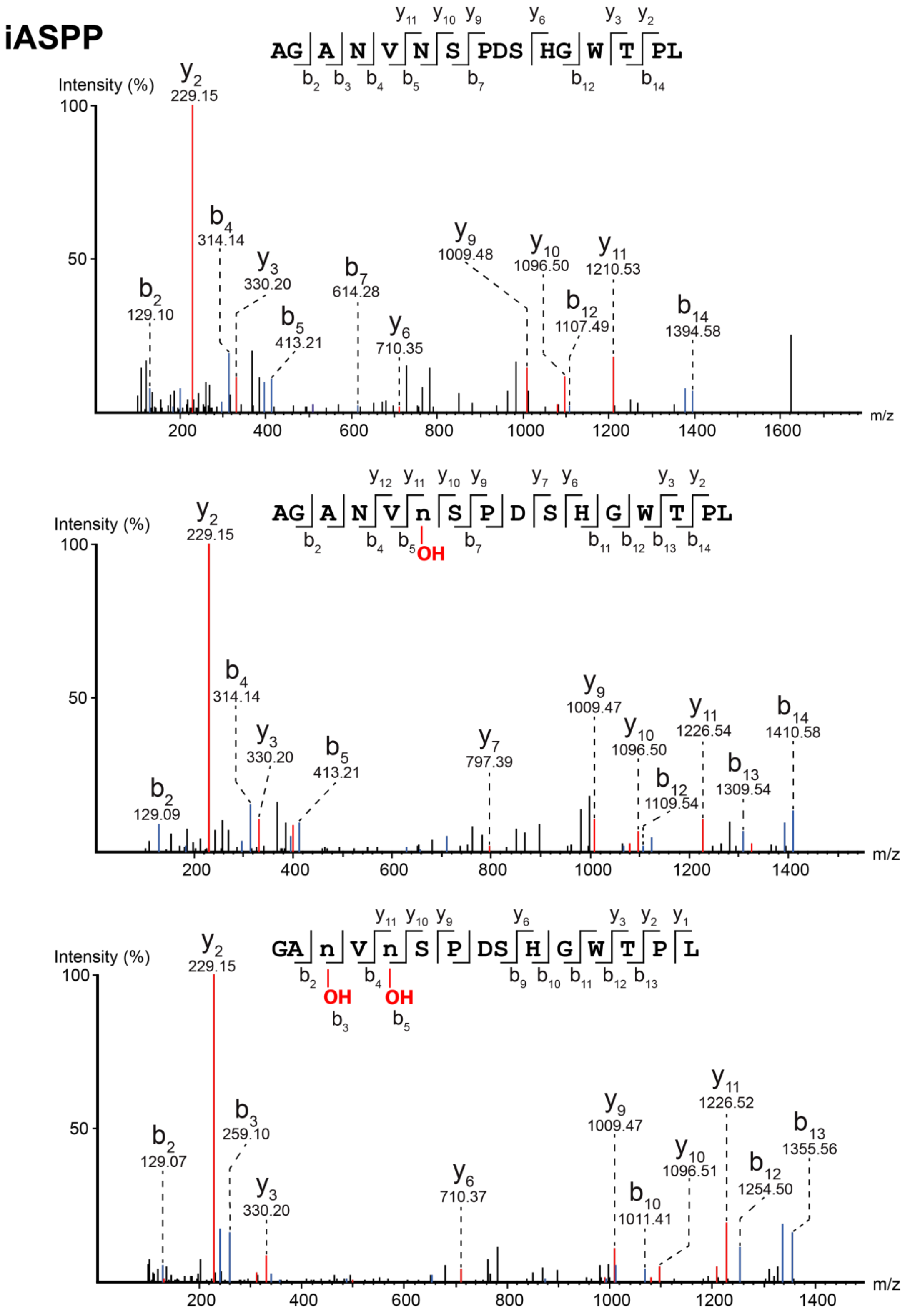

(Figure continues on next page)

iASPP

AG|A|N|V|N|S|PDS|HG|W|T|PL

#	b	b-H2O	b-NH3	b (2+)	Seq	y	y-H2O	y-NH3	y (2+)	#
1	72.04	54.03	55.02	36.52	A					16
2	129.10	111.06	112.04	65.03	G	1551.72	1533.71	1534.69	776.36	15
3	200.10	182.09	183.11	100.55	A	1494.70	1476.69	1477.67	747.85	14
4	314.14	296.14	297.12	157.57	N	1423.66	1405.65	1406.63	712.33	13
5	413.21	395.20	396.19	207.11	V	1309.62	1291.61	1292.59	655.31	12
6	527.26	509.25	510.22	264.13	N	1210.53	1192.54	1193.52	605.77	11
7	614.28	596.28	597.26	307.64	S	1096.50	1078.50	1079.46	548.75	10
8	711.34	693.33	694.32	356.17	P	1009.48	991.46	992.45	505.24	9
9	826.37	808.36	809.34	413.68	D	912.42	894.41	895.39	456.71	8
10	913.40	895.39	896.37	457.20	S	797.39	779.38	780.37	399.20	7
11	1050.46	1032.45	1033.43	525.73	H	710.35	692.35	693.34	355.68	6
12	1107.49	1089.47	1090.45	554.24	G	573.30	555.29	556.28	287.15	5
13	1293.56	1275.55	1276.53	647.28	W	516.28	498.27	499.25	258.64	4
14	1394.58	1376.58	1377.58	697.80	T	330.20	312.19	313.18	165.60	3
15	1491.66	1473.65	1474.63	746.33	P	229.15	211.14	212.13	115.08	2
16					L	132.10	114.09	115.07	66.55	1

AG|A|N|V|n|S|P|D|S|H|G|W|T|PL
OH

#	b	b-H2O	b-NH3	b (2+)	Seq	y	y-H2O	y-NH3	y (2+)	#
1	72.04	54.03	55.02	36.52	A					16
2	129.09	111.06	112.04	65.03	G	1567.71	1549.70	1550.69	784.36	15
3	200.10	182.09	183.11	100.55	A	1510.69	1492.68	1493.67	755.85	14
4	314.14	296.14	297.12	157.57	N	1439.65	1421.64	1422.63	720.33	13
5	413.21	395.20	396.19	207.11	V	1325.62	1307.60	1308.59	663.31	12
6	543.25	525.24	526.23	272.13	N(+15.99)	1226.54	1208.53	1209.52	613.77	11
7	630.28	612.27	613.26	315.64	S	1096.49	1078.50	1079.44	548.75	10
8	727.34	709.33	710.35	364.17	P	1009.47	991.46	992.45	505.24	9
9	842.36	824.35	825.34	421.68	D	912.42	894.41	895.39	456.71	8
10	929.40	911.39	912.37	465.20	S	797.39	779.38	780.37	399.20	7
11	1066.45	1048.44	1049.43	533.73	H	710.35	692.35	693.34	355.68	6
12	1123.47	1105.47	1106.46	562.24	G	573.30	555.29	556.28	287.15	5
13	1309.54	1291.55	1292.53	655.24	W	516.28	498.27	499.25	258.64	4
14	1410.58	1392.59	1393.57	705.80	T	330.20	312.19	313.18	165.60	3
15	1507.66	1489.65	1490.63	754.33	P	229.15	211.14	212.13	115.08	2
16					L	132.10	114.09	115.07	66.55	1

GA|n|V|n|S|P|DS|H|G|W|T|P|L
OH OH

#	b	b-H2O	b-NH3	b (2+)	Seq	y	y-H2O	y-NH3	y (2+)	#
1	58.03	40.02	41.00	29.51	G					15
2	129.07	111.06	112.04	65.03	A	1526.69	1508.68	1509.66	763.84	14
3	259.10	241.09	242.08	130.05	N(+15.99)	1455.65	1437.64	1438.62	728.32	13
4	358.17	340.16	341.15	179.59	V	1325.61	1307.60	1308.59	663.31	12
5	488.21	470.20	471.18	244.61	N(+15.99)	1226.52	1208.51	1209.49	613.77	11
6	575.24	557.23	558.22	288.12	S	1096.51	1078.50	1079.46	548.75	10
7	672.30	654.25	655.27	336.65	P	1009.47	991.47	992.45	505.24	9
8	787.32	769.31	770.30	394.16	D	912.42	894.41	895.39	456.71	8
9	874.34	856.34	857.33	437.68	S	797.39	779.38	780.37	399.20	7
10	1011.41	993.40	994.39	506.21	H	710.37	692.35	693.34	355.68	6
11	1068.43	1050.42	1051.41	534.72	G	573.30	555.29	556.28	287.15	5
12	1254.50	1236.50	1237.49	627.76	W	516.28	498.27	499.23	258.64	4
13	1355.56	1337.55	1338.53	678.28	T	330.20	312.17	313.18	165.60	3
14	1452.61	1434.60	1435.59	726.81	P	229.15	211.14	212.13	115.08	2
15					L	132.10	114.09	115.07	66.55	1

Figure S7 FIH catalysed hydroxylation of recombinant iASPP (625-828) protein. LC-MS/MS of recombinant iASPP (625-828) incubated with FIH. The spectrum shows fragments from the elastase catalysed digestion of iASPP providing evidence for hydroxylation at Asp-687 and Asp-685. Conditions: 1 μM iASPP, 0.5 μM FIH, 1 mM sodium ascorbate, 1 mM 2OG disodium salt, 200 μM, iron(II) ammonium sulfate, 50 mM Tris-HCl pH 7.5, incubation for 3 h at 37 °C.

ASPP1

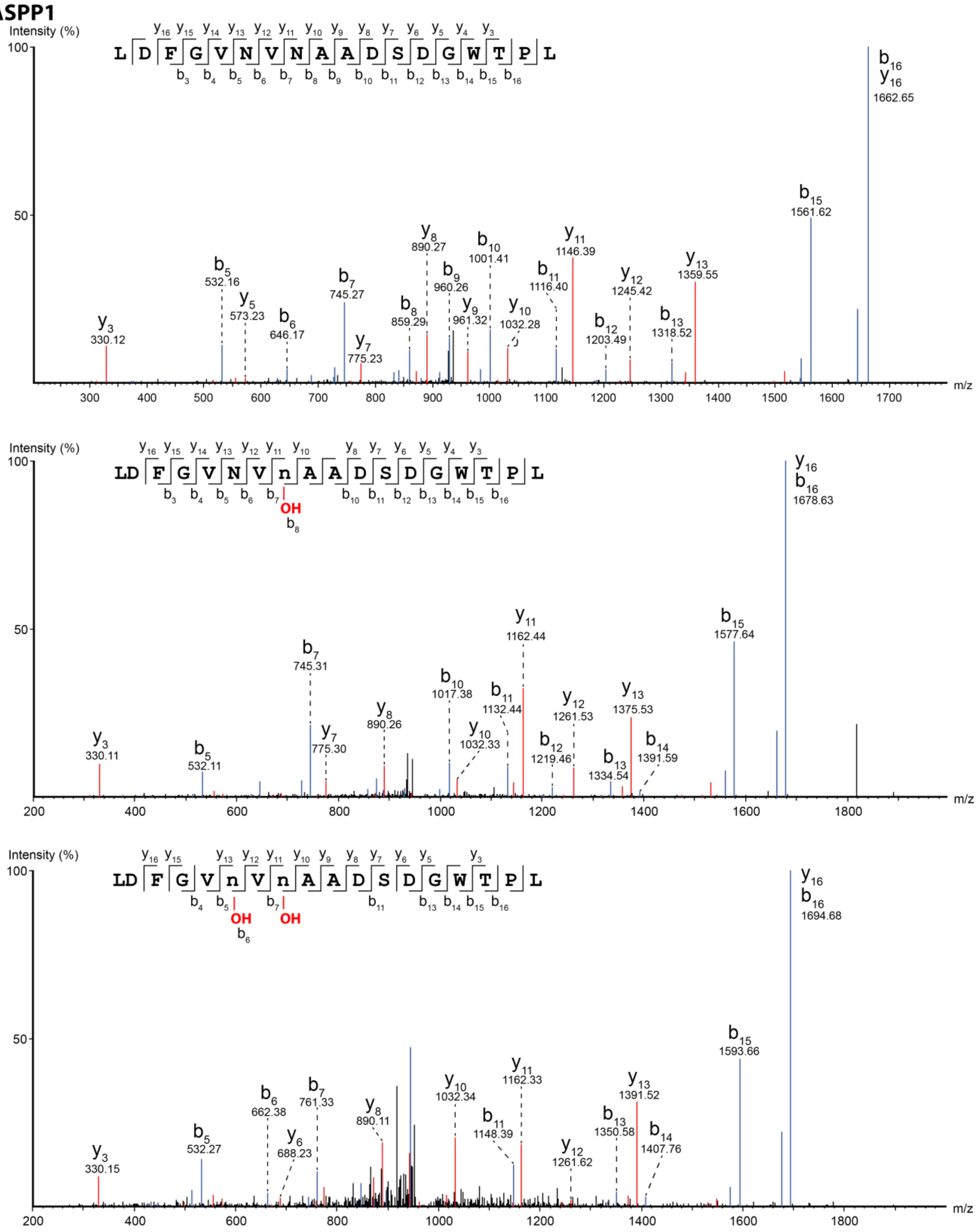

(Figure continues on next page)

ASPP1

L D F G V N V N A A D S D G W T P L

#	b	b-H2O	b-NH3	b (2+)	Seq	y	y-H2O	y-NH3	y (2+)	#
1	114.09	96.08	97.06	57.55	L					18
2	229.12	211.11	212.09	115.06	D	1777.80	1759.79	1760.78	889.62	17
3	376.22	358.30	359.16	188.59	F	1662.65	1644.67	1645.75	832.19	16
4	433.07	415.25	416.18	217.10	G	1515.73	1497.62	1498.59	758.26	15
5	532.16	514.13	515.25	266.64	V	1458.49	1440.68	1441.66	729.84	14
6	646.17	628.15	629.26	323.66	N	1359.55	1341.57	1342.54	680.31	13
7	745.27	727.28	728.20	373.05	V	1245.42	1227.56	1228.08	623.29	12
8	859.29	841.42	842.04	430.22	N	1146.39	1128.50	1129.48	573.75	11
9	930.26	912.45	913.40	465.73	A	1032.28	1014.35	1015.53	517.20	10
10	1001.41	983.46	984.45	501.25	A	961.32	943.42	944.38	481.16	9
11	1116.40	1098.52	1099.45	558.39	D	890.27	872.42	873.36	446.07	8
12	1203.49	1185.46	1186.62	602.34	S	775.23	757.11	758.26	388.18	7
13	1318.52	1300.74	1301.56	659.80	D	688.25	670.32	671.32	344.67	6
14	1375.44	1357.60	1358.59	688.25	G	573.23	555.30	556.28	287.15	5
15	1561.62	1543.50	1544.55	781.35	W	516.34	498.27	499.31	258.64	4
16	1662.65	1644.67	1645.71	832.19	T	330.12	312.42	313.18	165.60	3
17	1759.79	1741.78	1742.77	880.52	P	229.15	211.14	212.13	115.08	2
18					L	132.10	114.09	115.07	66.55	1

LD F G V N V n A A D S D G W T P L

OH

#	b	b-H2O	b-NH3	b (2+)	Seq	y	y-H2O	y-NH3	y (2+)	#
1	114.09	96.08	97.06	57.55	L					18
2	229.12	211.11	212.09	115.06	D	1793.80	1775.79	1776.77	897.40	17
3	376.12	358.18	359.16	188.59	F	1678.63	1660.62	1661.74	839.89	16
4	433.23	415.20	416.09	217.10	G	1531.66	1513.61	1514.68	766.27	15
5	532.11	514.26	515.04	266.64	V	1474.51	1456.79	1457.65	737.84	14
6	646.25	628.14	629.17	323.66	N	1375.53	1357.55	1358.59	688.22	13
7	745.31	727.29	728.28	373.19	V	1261.53	1243.55	1244.54	631.20	12
8	875.40	857.22	858.90	438.21	N(+15.99)	1162.44	1144.39	1145.47	581.75	11
9	946.46	928.21	929.22	473.73	A	1032.33	1014.45	1015.44	516.73	10
10	1017.38	999.44	1000.47	509.01	A	961.43	943.27	944.40	481.06	9
11	1132.44	1114.06	1115.50	566.79	D	890.26	872.16	873.36	446.17	8
12	1219.46	1201.62	1202.37	610.74	S	775.30	757.39	758.34	388.18	7
13	1334.54	1316.61	1317.56	667.79	D	688.22	670.32	671.23	344.67	6
14	1391.59	1373.60	1374.58	696.30	G	573.21	555.40	556.28	287.15	5
15	1577.64	1559.72	1560.66	789.67	W	516.18	498.27	499.72	258.64	4
16	1678.63	1660.62	1661.71	839.87	T	330.11	312.12	313.18	165.60	3
17	1775.79	1757.78	1758.76	888.41	P	229.15	211.14	212.13	115.08	2
18					L	132.10	114.09	115.07	66.55	1

LD F G V n V n A A D S D G W T P L

OH OH

#	b	b-H2O	b-NH3	b (2+)	Seq	y	y-H2O	y-NH3	y (2+)	#
1	114.09	96.08	97.06	57.55	L					18
2	229.12	211.11	212.09	115.06	D	1809.79	1791.78	1792.77	905.40	17
3	376.19	358.18	359.16	188.59	F	1694.68	1676.68	1677.74	847.93	16
4	433.35	415.20	416.18	217.10	G	1547.74	1529.69	1530.81	774.28	15
5	532.27	514.18	515.25	266.64	V	1490.68	1472.67	1473.65	745.84	14
6	662.38	644.01	645.29	331.66	N(+15.99)	1391.52	1373.60	1374.58	696.30	13
7	761.33	743.75	744.54	381.08	V	1261.62	1243.56	1244.28	631.28	12
8	891.42	873.41	874.61	446.02	N(+15.99)	1162.33	1144.49	1145.10	581.75	11
9	962.46	944.34	945.43	481.73	A	1032.34	1014.80	1015.44	516.73	10
10	1033.50	1015.44	1016.25	517.33	A	961.48	943.37	944.34	481.21	9
11	1148.39	1130.51	1131.50	574.76	D	890.11	872.18	873.36	446.02	8
12	1235.55	1217.54	1218.53	618.28	S	775.27	757.35	758.45	388.18	7
13	1350.58	1332.87	1333.55	675.79	D	688.23	670.32	671.74	344.67	6
14	1407.76	1389.59	1390.58	704.30	G	573.22	555.18	556.28	287.15	5
15	1593.66	1575.49	1576.66	797.00	W	516.28	498.27	499.22	258.64	4
16	1694.68	1676.68	1677.70	847.93	T	330.15	312.19	313.18	165.60	3
17	1791.78	1773.77	1774.76	896.84	P	229.15	211.14	212.13	115.08	2
18					L	132.10	114.09	115.07	66.55	1

Figure S8 FIH catalysed hydroxylation of recombinant ASPP1 (883-1090) protein. LC-MS/MS of recombinant ASPP (883-1090) incubated with FIH. The spectrum shows fragments from the elastase catalysed digestion of ASPP1 providing evidence for hydroxylation at Asp-948 and Asp-946. Conditions: 1 μM iASPP, 0.5 μM FIH, 1 mM sodium ascorbate, 1 mM 2OG disodium salt, 200 μM, iron(II) ammonium sulfate, 50 mM Tris-HCl pH 7.5, incubation for 3 h at 37 °C.

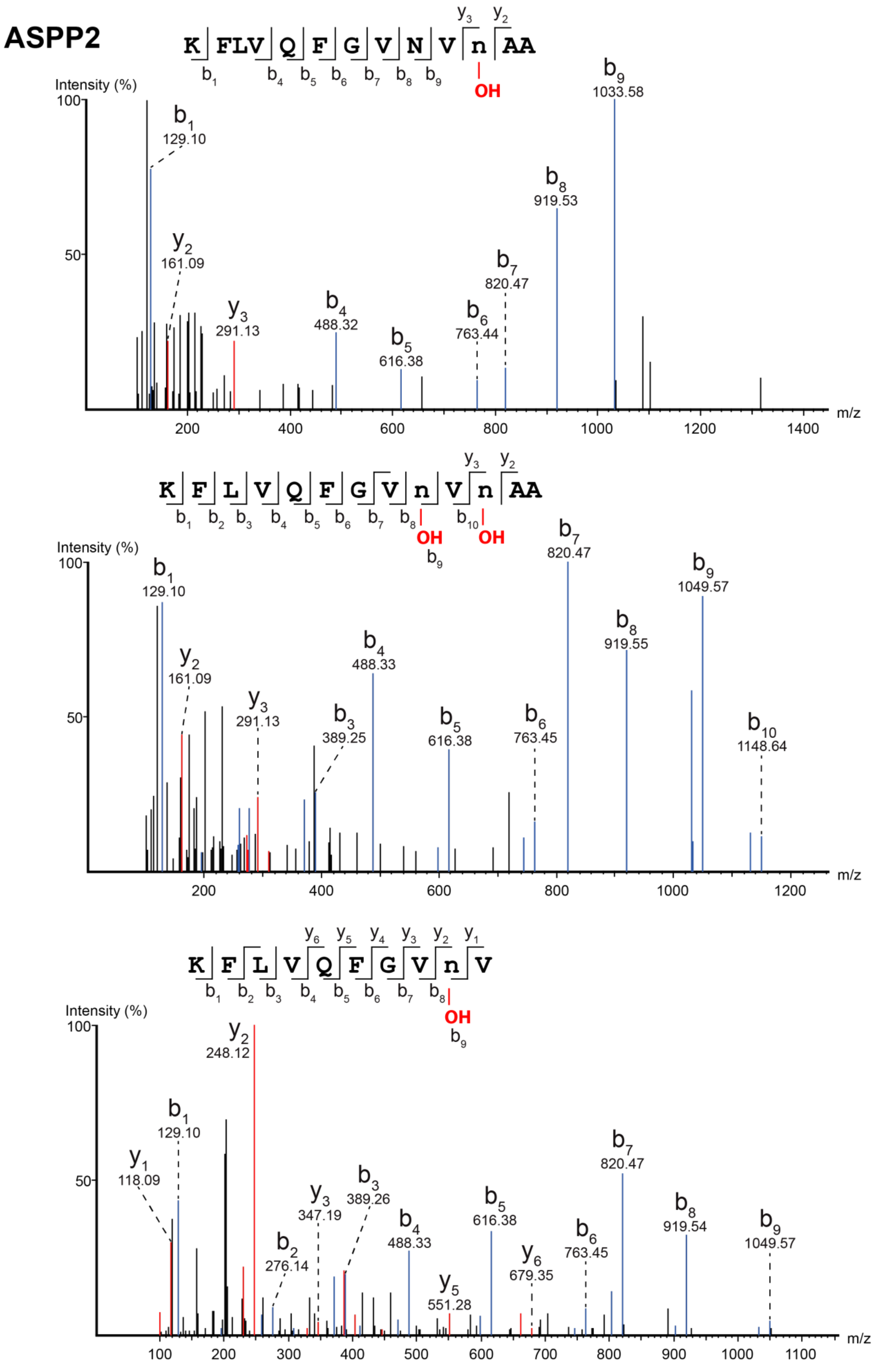

(Figure continues on next page)

ASPP2

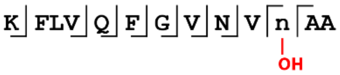

#	b	b-H2O	b-NH3	b (2+)	Seq	y	y-H2O	y-NH3	y (2+)	#
1	129.10	111.09	112.08	65.05	K					13
2	276.17	258.16	259.14	138.59	F	1294.68	1276.67	1277.65	647.84	12
3	389.26	371.24	372.23	195.13	L	1147.61	1129.60	1130.58	574.31	11
4	488.32	470.31	471.30	244.66	V	1034.53	1016.52	1017.50	517.76	10
5	616.38	598.37	599.36	308.69	Q	935.46	917.45	918.43	468.23	9
6	763.44	745.44	746.42	382.23	F	807.40	789.39	790.37	404.20	8
7	820.47	802.46	803.45	410.74	G	660.33	642.32	643.30	330.67	7
8	919.53	901.53	902.51	460.27	V	603.31	585.30	586.28	302.15	6
9	1033.58	1015.57	1016.56	517.29	N	504.24	486.23	487.21	252.62	5
10	1132.65	1114.64	1115.62	566.83	V	390.20	372.19	373.17	195.60	4
11	1262.69	1244.68	1245.66	631.84	N(+15.99)	291.13	273.12	274.10	146.06	3
12	1333.73	1315.72	1316.70	667.36	A	161.09	143.08	144.07	81.05	2
13					A	90.05	72.04	73.03	45.53	1

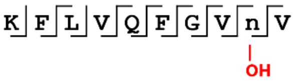

#	b	b-H2O	b-NH3	b (2+)	Seq	y	y-H2O	y-NH3	y (2+)	#
1	129.10	111.09	112.08	65.05	K					10
2	276.14	258.16	259.11	138.59	F	1038.56	1020.55	1021.53	519.78	9
3	389.26	371.25	372.23	195.11	L	891.49	873.48	874.47	446.28	8
4	488.33	470.31	471.30	244.66	V	778.41	760.40	761.38	389.70	7
5	616.38	598.37	599.36	308.70	Q	679.35	661.33	662.35	340.17	6
6	763.45	745.45	746.43	382.23	F	551.28	533.27	534.26	276.14	5
7	820.47	802.46	803.45	410.74	G	404.23	386.21	387.20	202.61	4
8	919.54	901.53	902.51	460.27	V	347.19	329.18	330.21	174.10	3
9	1049.57	1031.56	1032.55	525.29	N(+15.99)	248.12	230.11	231.10	124.56	2
10					V	118.09	100.08	101.07	59.54	1

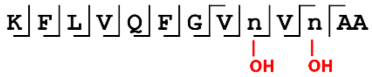

#	b	b-H2O	b-NH3	b (2+)	Seq	y	y-H2O	y-NH3	y (2+)	#
1	129.10	111.09	112.08	65.05	K					13
2	276.17	258.16	259.11	138.59	F	1310.67	1292.66	1293.65	655.84	12
3	389.25	371.25	372.23	195.11	L	1163.61	1145.59	1146.58	582.30	11
4	488.33	470.31	471.30	244.66	V	1050.52	1032.51	1033.49	525.76	10
5	616.38	598.37	599.37	308.69	Q	951.45	933.44	934.43	476.23	9
6	763.45	745.43	746.42	382.23	F	823.39	805.38	806.37	412.20	8
7	820.47	802.46	803.45	410.74	G	676.33	658.32	659.30	338.66	7
8	919.55	901.53	902.51	460.27	V	619.30	601.29	602.28	310.14	6
9	1049.57	1031.56	1032.56	525.29	N(+15.99)	520.24	502.23	503.21	260.62	5
10	1148.64	1130.61	1131.62	574.82	V	390.20	372.19	373.17	195.60	4
11	1278.68	1260.67	1261.66	639.84	N(+15.99)	291.13	273.12	274.10	146.06	3
12	1349.72	1331.71	1332.69	675.36	A	161.09	143.08	144.07	81.05	2
13					A	90.05	72.04	73.03	45.53	1

Figure S9 FIH catalysed hydroxylation of recombinant ASPP2 (889-1128) protein. LC-MS/MS of recombinant ASPP2 (889-1128) incubated with FIH. The spectrum shows fragments from the elastase catalysed digestion of ASPP2 providing evidence for hydroxylation of Asp-986 and Asp-984. Note the elastase digest yields apparently hydroxylated peptides not covering the canonical FIH site supporting a second hydroxylation. Conditions: 1 μM iASPP, 0.5 μM FIH, 1 mM sodium ascorbate, 1 mM 2OG disodium salt, 200 μM iron(II) ammonium sulfate, 50 mM Tris-HCl pH 7.5, incubation for 3 h at 37 °C.

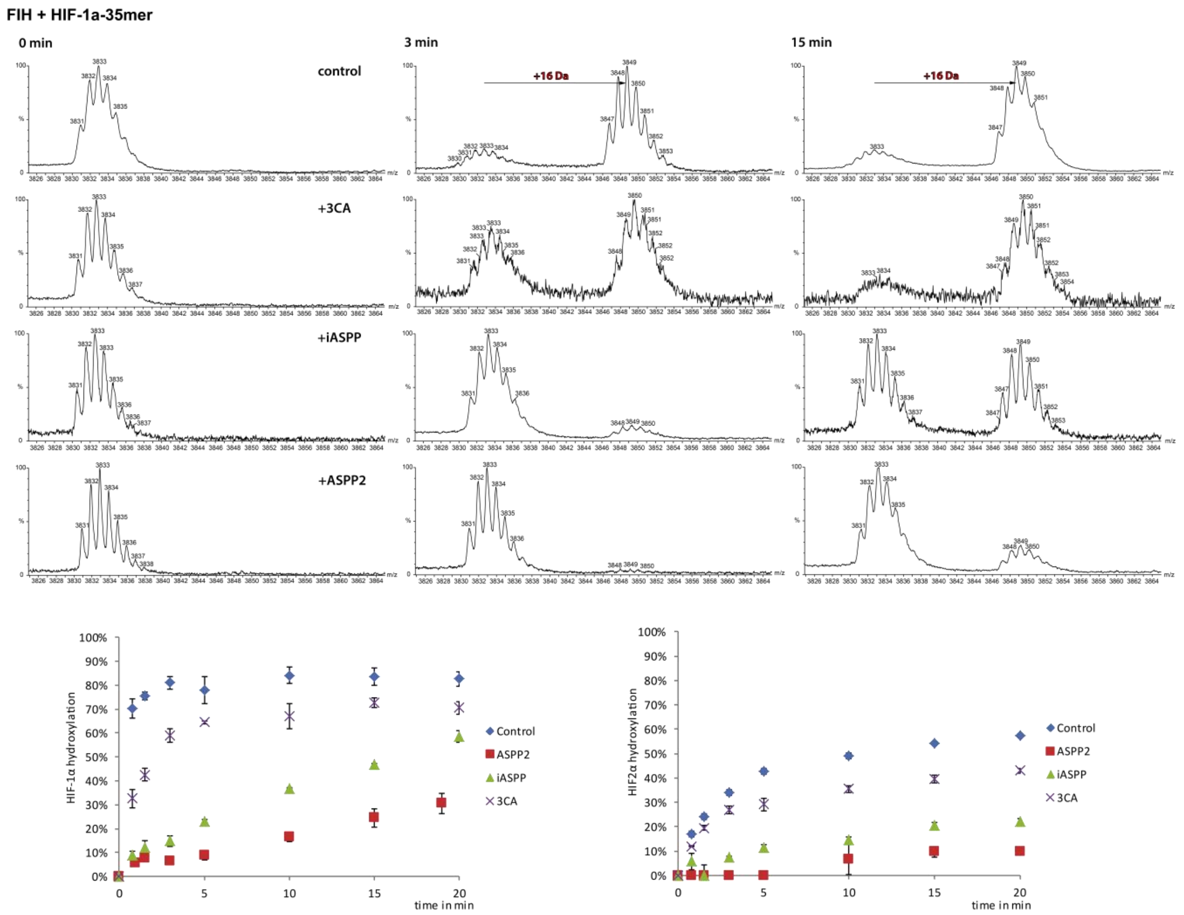

Figure S10 ASPP proteins compete with HIF-α for FIH catalysed reactions. Top: representative MALDI MS spectra for FIH mediated hydroxylation of HIF-1α (789-822) in the presence of ARD proteins. **Bottom:** Time course of FIH mediated hydroxylation of HIF-1α (789-822) and HIF-2α (832-866) in the presence of ARD proteins (ASPP2889-1128-Avi-His_6 and iASPP625-828-Avi-His_6, 3CA sequence according to Hardy et al. (1)). Conditions: 4 μM FIH, 100 μM HIF-α 35mer, 100 μM ARD protein, 1 mM sodium ascorbate, 1 mM 2OG disodium salt, 200 μM Fe(II), 50 mM Tris-HCl pH 7.5, with incubation for the indicated times at 37 °C.

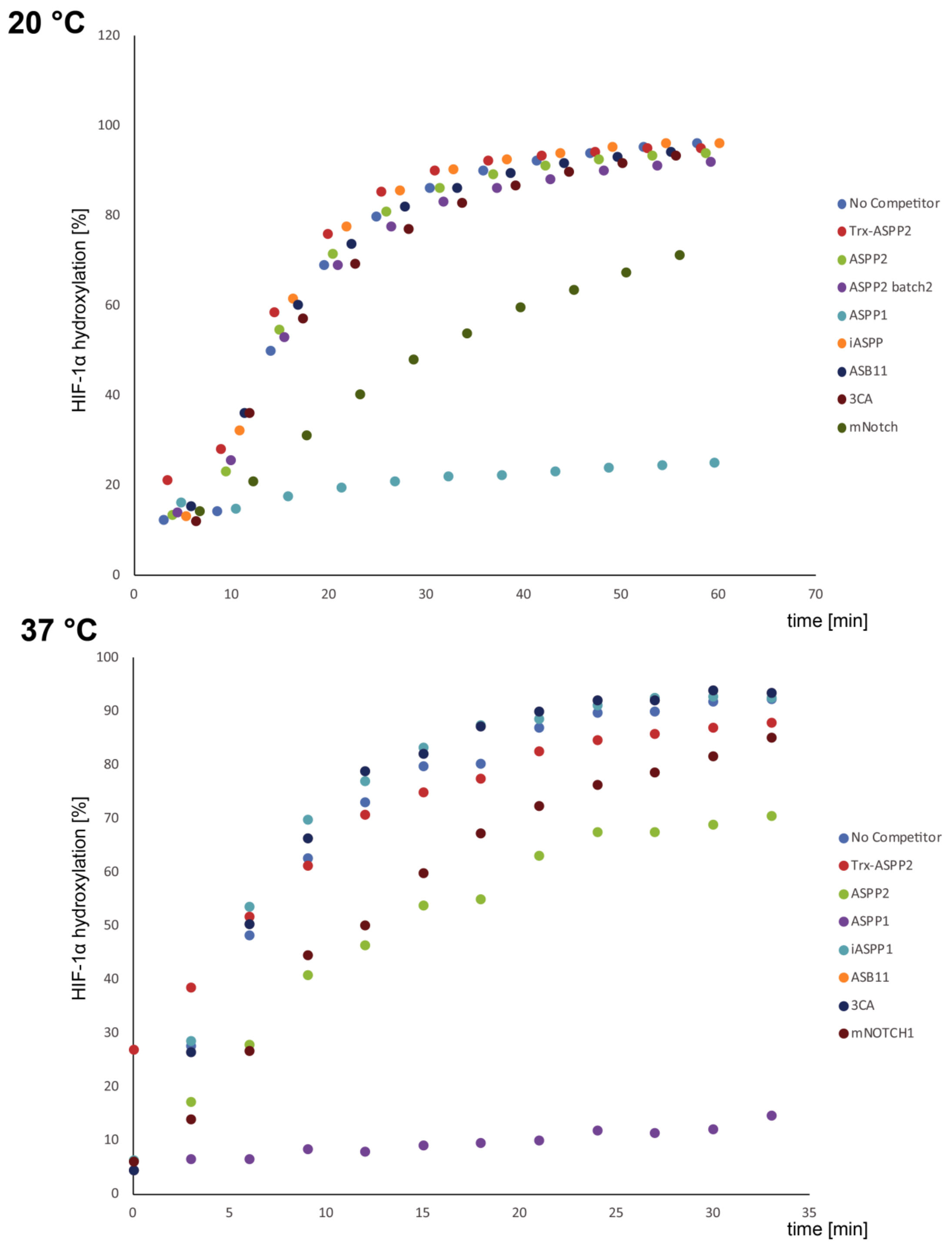

Figure S11 Competition of ARD proteins with HIF-1α for recombinant isolated FIH activity. Reaction conditions: 0.1 μM FIH, 100 μM sodium ascorbate, 10 μM 2OG disodium salt, 10 μM Fe(II), 50 mM Tris-HCl pH 7.5, 50 mM NaCl, 5 μM HIF-1α (789-822), 1 μM ankyrin repeat domain (ARD) proteins, incubated at ambient temperature. ARD constructs used: ASPP2 (889-1128), ASPP1 (883-1090), iASPP (625-828), ASB11 (64-287), mNotch1 (1899-2106) (3). Note the change in efficiency of inhibition of HIF-1α inhibition changes with temperature; the reason for this is unclear, but may in part relate to temperature dependent conformational changes or temperature dependent differences in O_2 solubility.

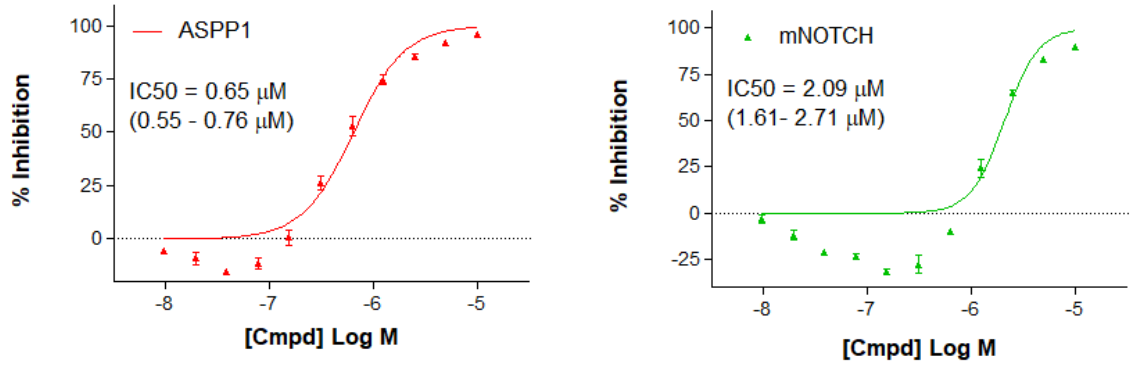

Figure S12 Half maximal inhibitory concentration (IC_{50}) measurements of ASPP1 (883-1090) and mNOTCH1 (1899-2106) competition for FIH mediated HIF-1α (789-822) hydroxylation. Conditions: 0.1 µM FIH, 100 µM sodium ascorbate, 10 µM 2OG disodium salt, 10 µM Fe(II), 50 mM Tris-HCl pH 7.5, 50 mM NaCl, incubated at ambient temperature. IC_{50}'s were determined from an 11-point titration and each point represents the mean ± SD of three determinations. The data represent the IC_{50} ± lower and upper 95% confidence intervals (in parentheses).

A

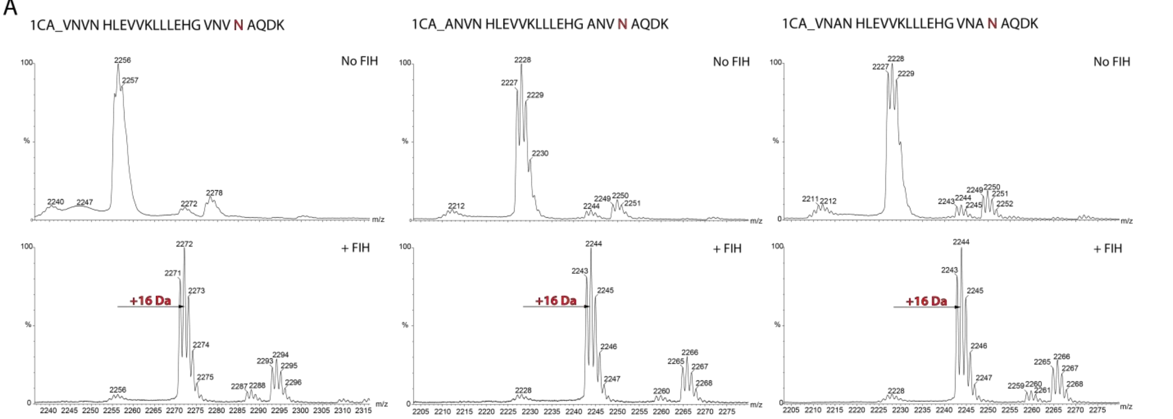

B

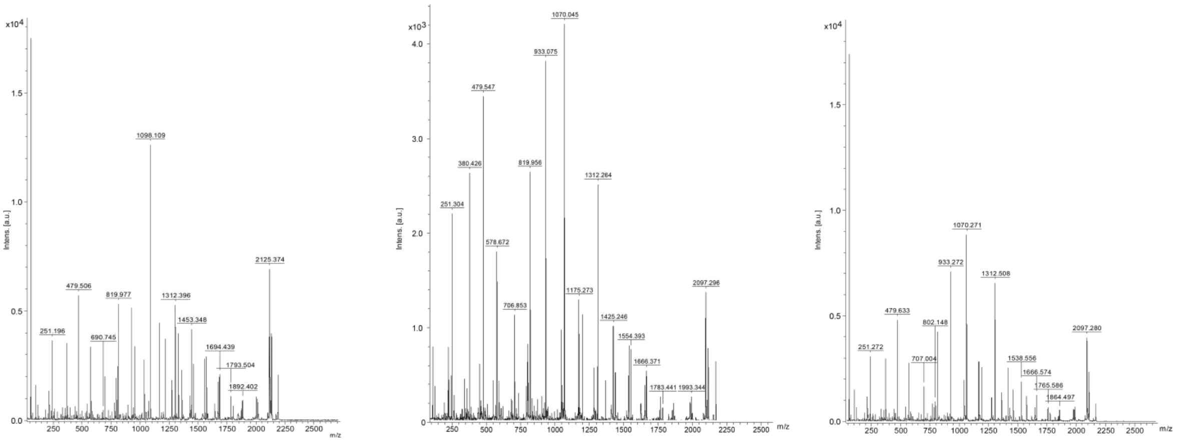

#	b	a	Seq	y	#
1			H		20
2	251.20		L	2134.46	19
3	380.35		E	2021.45	18
4	479.51		V	1892.40	17
5	578.65		V	1793.5	16
6	706.88		K	1694.44	15
7	819.98		L	1566.42	14
8	933.14		L	1453.35	13
9	1046.26		L		12
10	1175.30		E	1227.20	11
11	1312.40	1284.34	H	1098.11	10
12	1369.42	1340.28	G	961.00	9
13	1468.45	1440.45	V		8
14	1582.47		N	804.93	7
15	1681.45	1653.52	V		6
16	1811.47		N (OH)		5
17	1882.40		A		4
18	2010.44	1854.58	Q		3
19	2125.37		D		2
20			K		1

#	b	a	Seq	y	#
1			H		20
2	251.3		L	2106.35	19
3	380.43		E	1993.34	18
4	479.55	451.54	V	1864.35	17
5	578.67	550.69	V	1765.38	16
6	706.85		K	1666.37	15
7	819.96	791.91	L	1538.32	14
8	933.07		L	1215.25	13
9	1046.19		L	1312.26	12
10	1175.27		E	1199.12	11
11		1284.34	H	1070.05	10
12			G		9
13	1440.32	1412.37	A		8
14	1554.39		N	1369.33	7
15	1653.38		V		6
16	1783.44		N (OH)	591.64	5
17	1854.43		A		4
18	1982.41		Q		3
19	2097.3		D		2
20			K		1

#	b	a	Seq	y	#
1			H		20
2	251.21		L	2106.34	19
3	380.46		E	1993.42	18
4	479.63		V	1864.50	17
5	578.81		V		16
6	707.00		K	1765.59	15
7	820.18		L	1538.56	14
8	933.27		L	1425.52	13
9	1046.45		L		12
10	1175.51		E	1199.36	11
11	1312.51	1284.63	H	1070.27	10
12	1369.60		G		9
13	1468.62	1440.71	V		8
14	1582.63		N		7
15		1625.65	A		6
16	1783.51		N (OH)		5
17	1854.57		A		4
18	1982.52		Q		3
19	2097.28		D		2
20			K		1

Figure S13 FIH hydroxylation assays of consensus ankyrin repeat domain (1CA)(1) derived peptides bearing a "VNVN", "ANVN" or "VNAN" motif. A End point hydroxylation assays of 1CA_VNVN, 1CA_ANVN and 1CA_VNAN. **B** MALDI LIFT-TOF MS/MS analysis of hydroxylated 1CA derived peptides. Conditions: 50 μM peptide, 50 μM FIH, 1 mM ascorbate, 1 mM 2OG, 200 μM Fe(II), incubation for 30 min at 37 °C.

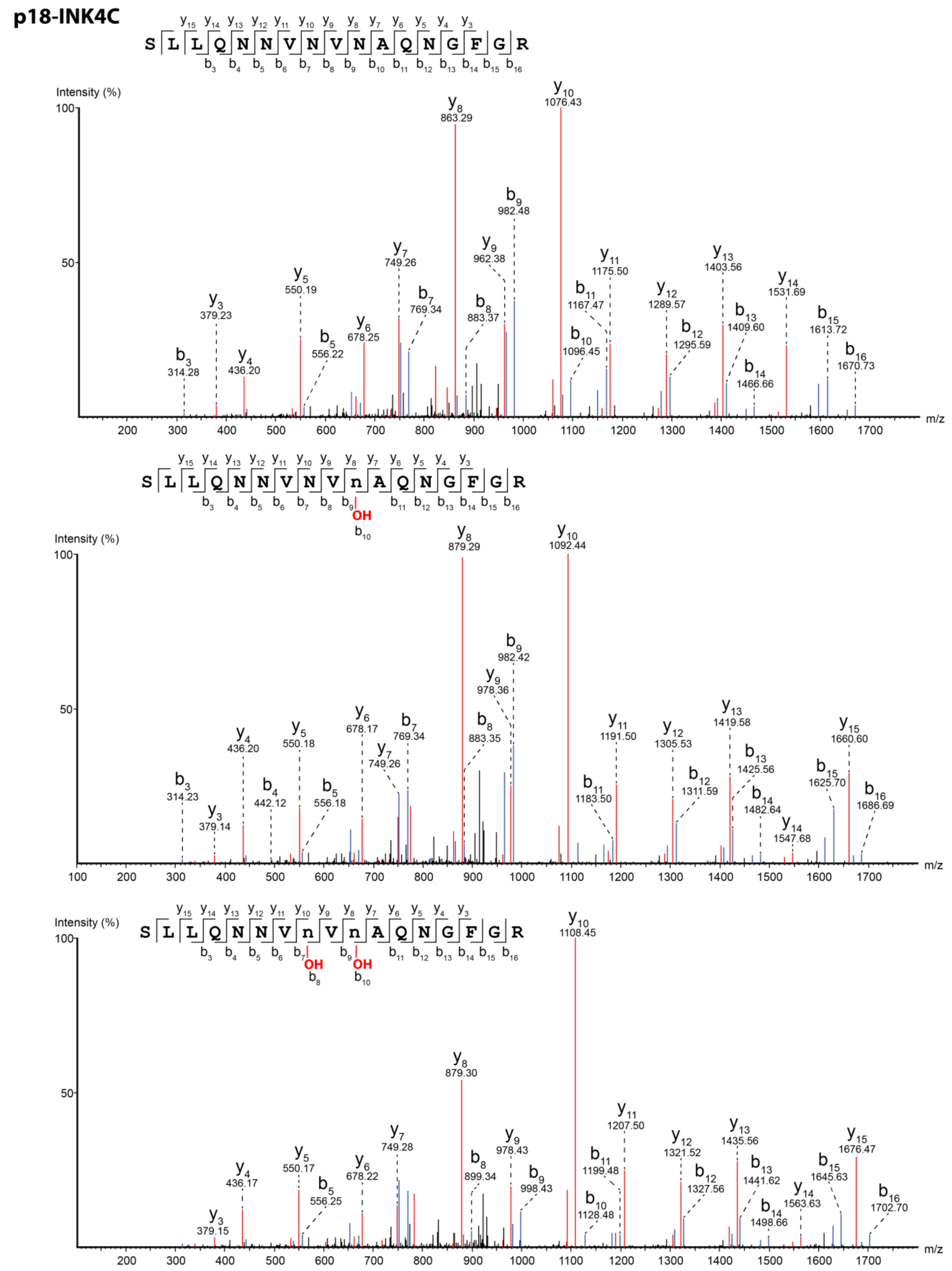

(Figure continues on next page)

p18-INK4C

S L L Q N N V N V N A Q N G F G R

#	b	b-H2O	b-NH3	b (2+)	Seq	y	y-H2O	y-NH3	y (2+)	#
1	88.04	70.03	71.01	44.52	S					17
2	201.12	183.11	184.10	101.06	L	1757.90	1739.89	1740.88	879.78	16
3	314.28	296.20	297.18	157.60	L	1644.82	1626.81	1628.00	822.77	15
4	442.20	424.37	425.24	221.63	Q	1531.69	1513.69	1514.64	766.37	14
5	556.22	538.18	539.22	278.65	N	1403.56	1385.76	1386.61	702.40	13
6	670.22	652.23	653.24	335.68	N	1289.57	1271.46	1272.52	645.32	12
7	769.34	751.34	752.32	385.21	V	1175.50	1157.70	1158.48	588.30	11
8	883.37	865.45	866.34	442.20	N	1076.43	1058.41	1059.46	539.22	10
9	982.48	964.52	965.39	491.77	V	962.38	944.42	945.39	481.67	9
10	1096.45	1078.56	1079.40	548.79	N	863.29	845.33	846.28	432.21	8
11	1167.47	1149.50	1150.51	584.31	A	749.26	731.27	732.22	375.66	7
12	1295.59	1277.62	1278.59	648.23	Q	678.25	660.21	661.21	340.09	6
13	1409.60	1391.62	1392.58	705.36	N	550.19	532.28	533.15	275.64	5
14	1466.66	1448.71	1449.59	734.27	G	436.20	418.24	419.29	218.62	4
15	1613.72	1595.67	1596.68	807.40	F	379.23	360.81	362.11	190.10	3
16	1670.73	1652.70	1653.69	835.91	G	232.14	214.13	215.11	116.57	2
17					R	175.12	157.11	158.09	88.06	1

S L L Q N N V N V n A Q N G F G R
OH

#	b	b-H2O	b-NH3	b (2+)	Seq	y	y-H2O	y-NH3	y (2+)	#
1	88.04	70.03	71.01	44.52	S					17
2	201.12	183.11	184.10	101.06	L	1773.90	1755.89	1756.87	887.42	16
3	314.23	296.23	297.18	157.60	L	1660.60	1642.80	1643.70	830.91	15
4	442.12	424.22	425.35	221.63	Q	1547.68	1529.65	1530.75	774.47	14
5	556.18	538.30	539.29	278.65	N	1419.58	1401.45	1402.52	710.38	13
6	670.20	652.19	653.19	335.68	N	1305.53	1287.62	1288.61	653.19	12
7	769.34	751.23	752.25	385.21	V	1191.50	1173.58	1174.48	596.36	11
8	883.35	865.35	866.28	442.12	N	1092.44	1074.56	1075.40	546.76	10
9	982.42	964.38	965.39	492.24	V	978.36	960.41	961.32	489.74	9
10	1112.53	1094.56	1095.54	556.78	N(+15.99)	879.29	861.41	862.32	440.20	8
11	1183.50	1165.53	1166.58	592.30	A	749.26	731.18	732.22	375.12	7
12	1311.59	1293.57	1294.55	656.36	Q	678.17	660.15	661.25	340.11	6
13	1425.56	1407.58	1408.68	713.35	N	550.18	532.16	533.12	275.64	5
14	1482.64	1464.70	1465.69	741.87	G	436.20	418.22	419.23	218.62	4
15	1629.70	1611.82	1612.70	815.48	F	379.14	361.20	361.72	190.10	3
16	1686.69	1668.62	1669.67	844.29	G	232.14	214.13	215.11	116.57	2
17					R	175.12	157.11	158.09	88.06	1

S L L Q N N V n V n A Q N G F G R
OH OH

#	b	b-H2O	b-NH3	b (2+)	Seq	y	y-H2O	y-NH3	y (2+)	#
1	88.04	70.03	71.01	44.52	S					17
2	201.12	183.11	184.10	101.06	L	1789.89	1771.88	1772.87	895.59	16
3	314.22	296.16	297.18	157.60	L	1676.47	1658.67	1659.78	838.90	15
4	442.16	424.15	425.11	221.63	Q	1563.63	1545.61	1546.58	782.50	14
5	556.25	538.19	539.23	278.65	N	1435.56	1417.63	1418.65	718.36	13
6	670.29	652.26	653.21	335.68	N	1321.52	1303.59	1304.54	661.20	12
7	769.34	751.30	752.28	385.21	V	1207.50	1189.67	1190.44	604.28	11
8	899.34	881.45	882.33	450.36	N(+15.99)	1108.45	1090.36	1091.38	555.19	10
9	998.43	980.52	981.36	499.76	V	978.43	960.57	961.49	489.25	9
10	1128.48	1110.55	1111.54	564.78	N(+15.99)	879.30	861.32	862.30	440.20	8
11	1199.48	1181.49	1182.58	600.30	A	749.28	731.27	732.32	375.18	7
12	1327.56	1309.64	1310.63	664.33	Q	678.22	660.24	661.20	340.13	6
13	1441.62	1423.56	1424.53	721.03	N	550.17	532.08	533.07	275.64	5
14	1498.66	1480.66	1481.67	749.86	G	436.17	418.27	419.16	218.62	4
15	1645.63	1627.67	1628.69	823.40	F	379.15	361.20	362.44	190.10	3
16	1702.70	1684.77	1685.76	851.91	G	232.14	214.13	215.11	116.57	2
17					R	175.12	157.11	158.09	88.06	1

Figure S14 FIH catalysed hydroxylation of recombinant full-length p18-INK4C protein. LC-MS/MS analysis of recombinant full-length p18-INK4C incubated with FIH. The spectrum shows fragments from the elastase catalysed digestion of p18-INK4C, providing evidence for hydroxylation at Asp-32 and Asp-30. Conditions: 1 µM p18-INK4C, 0.5 µM FIH, 1 mM sodium ascorbate, 1 mM 2OG disodium salt, 200 µM, iron(II) ammonium sulfate, 50 mM Tris-HCl pH 7.5, incubation for 3 h at 37 °C.

ASB11

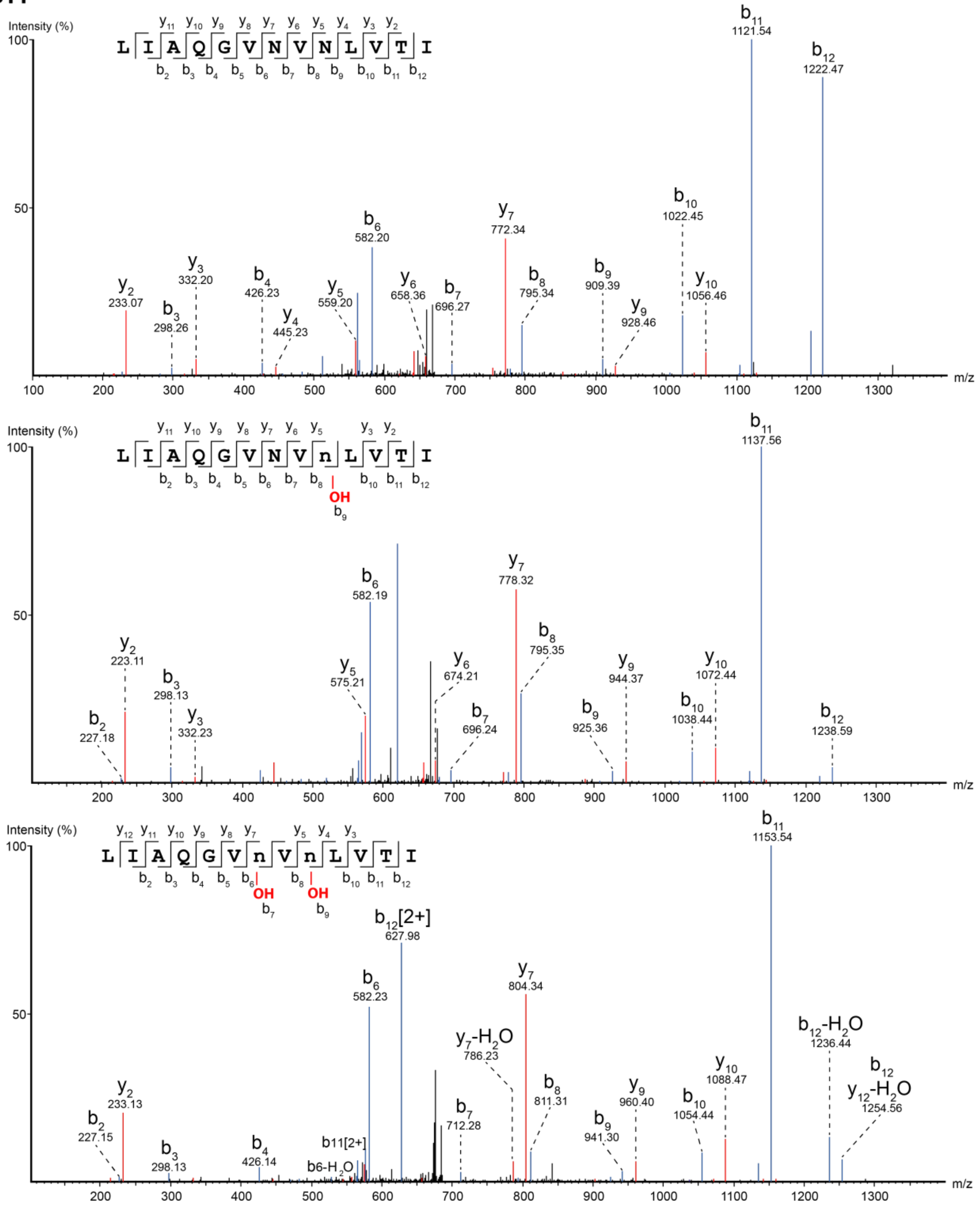

(Figure continues on next page)

ASB11

L I A Q G V N V N L V T I

#	b	b-H2O	b-NH3	b (2+)	Seq	y	y-H2O	y-NH3	y (2+)	#
1	114.09	96.08	97.06	57.55	L					13
2	227.16	209.17	210.15	114.09	I	1240.73	1222.47	1223.70	621.00	12
3	298.26	280.20	280.99	149.61	A	1127.50	1109.48	1110.73	564.22	11
4	426.23	408.26	409.32	213.64	Q	1056.46	1038.59	1039.37	529.09	10
5	483.22	465.28	466.14	242.15	G	928.46	910.54	911.52	464.77	9
6	582.20	564.22	565.09	291.68	V	871.30	853.12	854.50	436.26	8
7	696.27	678.39	679.38	348.70	N	772.34	754.26	755.43	386.73	7
8	795.34	777.16	778.19	397.98	V	658.36	640.48	641.24	329.71	6
9	909.39	891.29	892.49	455.11	N	559.20	541.33	542.41	280.17	5
10	1022.45	1004.66	1005.47	511.89	L	445.23	427.29	428.07	223.15	4
11	1121.54	1103.48	1104.52	561.39	V	332.20	314.29	315.38	166.61	3
12	1222.47	1204.73	1205.69	611.86	T	233.07	215.04	216.08	117.07	2
13					I	132.10	114.09	115.07	66.55	1

L I A Q G V N V n L V T I

OH

#	b	b-H2O	b-NH3	b (2+)	Seq	y	y-H2O	y-NH3	y (2+)	#
1	114.09	96.08	97.06	57.55	L					13
2	227.18	209.17	210.15	114.09	I	1256.72	1238.59	1239.69	629.03	12
3	298.13	280.20	281.19	149.61	A	1143.52	1125.55	1126.61	572.32	11
4	426.11	408.26	409.22	213.64	Q	1072.44	1054.29	1055.48	536.80	10
5	483.24	465.25	466.25	242.15	G	944.37	926.53	927.51	472.77	9
6	582.19	564.20	565.22	291.68	V	887.39	869.66	870.49	444.12	8
7	696.24	678.04	679.03	348.95	N	788.32	770.43	771.42	394.73	7
8	795.35	777.46	778.29	398.11	V	674.21	655.95	657.23	338.02	6
9	925.36	907.16	908.48	463.10	N(+15.99)	575.21	557.33	558.31	288.17	5
10	1038.44	1020.78	1021.47	519.87	L	445.30	427.29	428.28	223.15	4
11	1137.56	1119.39	1120.60	569.38	V	332.23	314.29	315.08	166.61	3
12	1238.59	1220.67	1221.68	619.94	T	233.11	215.10	216.12	117.07	2
13					I	132.10	114.09	115.07	66.55	1

L I A Q G V n V n L V T I

OH OH

#	b	b-H2O	b-NH3	b (2+)	Seq	y	y-H2O	y-NH3	y (2+)	#
1	114.09	96.08	97.06	57.55	L					13
2	227.15	209.36	210.15	114.09	I	1272.72	1254.56	1255.69	636.86	12
3	298.13	280.05	281.19	149.61	A	1159.48	1141.44	1142.60	580.32	11
4	426.14	408.35	409.36	214.13	Q	1088.47	1070.58	1071.47	544.64	10
5	483.13	464.90	466.28	242.11	G	960.40	942.53	943.51	481.20	9
6	582.23	564.15	565.20	291.68	V	903.43	885.58	886.49	452.27	8
7	712.28	694.39	695.37	356.70	N(+15.99)	804.34	786.23	787.42	403.10	7
8	811.31	793.35	794.44	406.60	V	674.41	656.40	657.07	338.14	6
9	941.30	923.50	924.43	470.92	N(+15.99)	575.22	557.20	558.31	288.17	5
10	1054.44	1036.42	1037.56	528.22	L	445.13	427.29	428.28	223.15	4
11	1153.54	1135.57	1136.63	577.31	V	332.14	314.21	315.11	166.61	3
12	1254.56	1236.44	1237.68	627.98	T	233.13	215.10	216.12	117.07	2
13					I	132.10	114.09	115.07	66.55	1

Figure S15 FIH catalysed hydroxylation of recombinant ASB11 (64-287) protein. LC-MS/MS analysis of recombinant full-length ASB11 (64-287) incubated with FIH. The spectrum shows fragments from the elastase catalysed digestion of ASB11 providing evidence for hydroxylation at Asp-92 and Asp-90. Conditions: 1 μM ASB11, 0.5 μM FIH, 1 mM sodium ascorbate, 1 mM 2OG disodium salt, 200 μM, iron(II) ammonium sulfate, 50 mM Tris-HCl pH 7.5, incubation for 3 h at 37 °C.

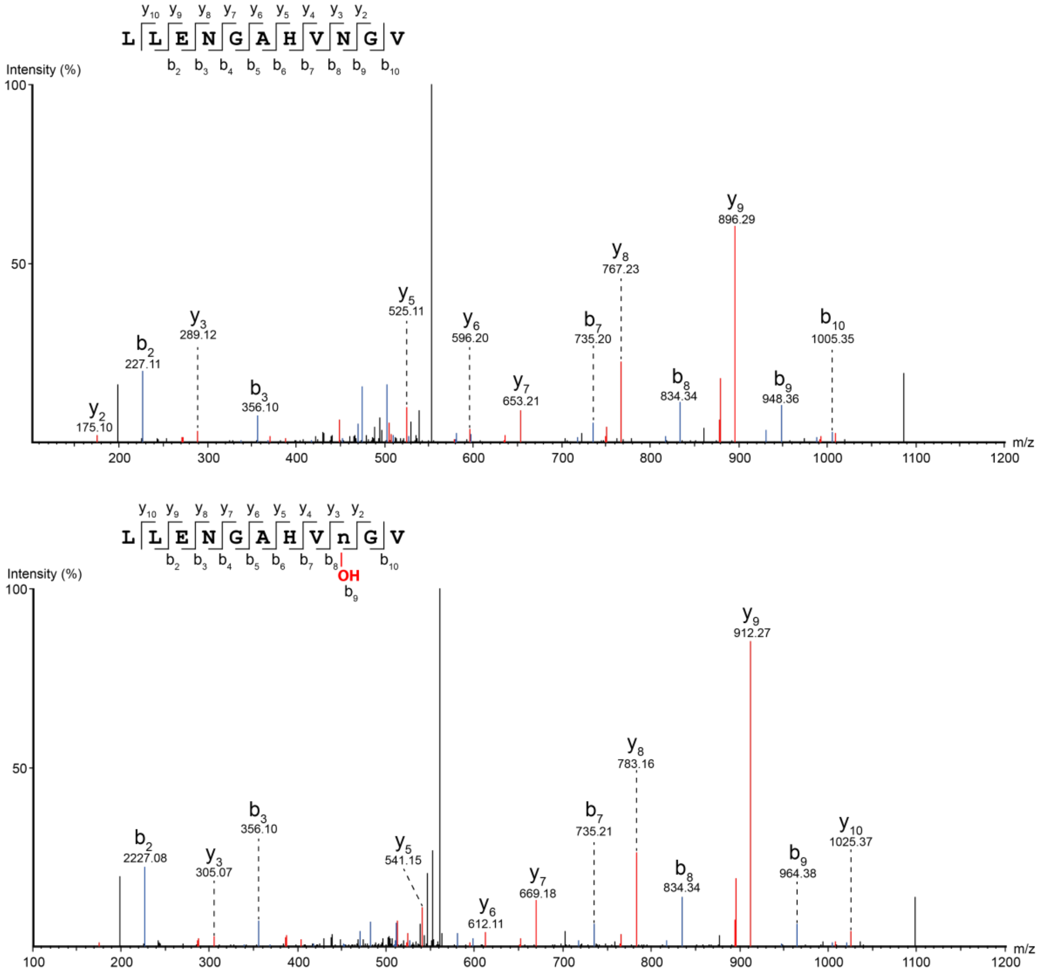

L L E N G A H V N G V

#	b	b-H2O	b-NH3	b (2+)	Seq	y	y-H2O	y-NH3	y (2+)	#
1	114.09	96.08	97.06	57.55	L					11
2	227.11	209.18	210.15	114.09	L	1009.35	991.37	992.38	505.32	10
3	356.10	338.23	339.19	178.61	E	896.29	878.26	879.21	448.83	9
4	470.14	452.29	453.23	235.63	N	767.23	749.22	750.19	384.38	8
5	527.12	509.27	510.12	264.14	G	653.21	635.22	636.26	327.15	7
6	598.10	580.20	581.12	299.24	A	596.20	578.28	579.18	298.66	6
7	735.20	717.32	718.28	368.31	H	525.11	507.27	508.09	263.14	5
8	834.34	816.44	817.29	417.92	V	388.22	370.11	371.07	194.61	4
9	948.36	930.37	931.35	474.83	N	289.12	271.09	272.10	145.08	3
10	1005.35	987.29	988.47	503.41	G	175.10	157.10	158.08	88.05	2
11					V	118.09	100.08	101.06	59.54	1

L L E N G A H V n G V (n: OH)

#	b	b-H2O	b-NH3	b (2+)	Seq	y	y-H2O	y-NH3	y (2+)	#
1	114.09	96.08	97.06	57.55	L					11
2	227.08	209.14	210.07	114.09	L	1025.37	1007.34	1008.40	513.26	10
3	356.10	338.17	339.16	178.99	E	912.27	894.26	895.29	456.71	9
4	470.14	452.08	453.10	235.63	N	783.16	765.14	766.24	392.32	8
5	527.18	509.15	510.11	263.94	G	669.18	651.09	652.17	335.17	7
6	598.21	580.35	581.18	299.66	A	612.11	594.59	595.24	306.32	6
7	735.21	717.14	718.14	368.21	H	541.15	523.06	524.08	271.14	5
8	834.34	816.44	817.38	417.73	V	404.04	386.12	387.10	202.21	4
9	964.38	946.47	947.39	482.76	N(+15.99)	305.07	287.11	288.09	153.07	3
10	1021.43	1003.50	1004.38	511.23	G	175.09	157.10	158.08	88.05	2
11					V	118.09	100.08	101.06	59.54	1

Figure S16 FIH catalysed hydroxylation of recombinant ASB11 (64-287) protein at Asn-125. LC-MS/MS analysis of recombinant full-length ASB11 (64-287) incubated with FIH. The spectrum shows fragments from the elastase catalysed digestion of ASB11 providing evidence for hydroxylation at Asp-125. Conditions: 1 μM ASB11, 0.5 μM FIH, 1 mM sodium ascorbate, 1 mM 2OG disodium salt, 200 μM, iron(II) ammonium sulfate, 50 mM Tris-HCl pH 7.5, incubation for 3 h at 37 °C.

Table S1 Ion tables for MS-MS spectra from MALDI LIFT-TOF-TOF analysis of the FIH hydroxylation site of the iASPP (670-693) peptide (see Figure S5).

iASPP 24mer

#	b	Seq	y	#
1		G		24
2		A		23
3	243.11	N	2291.09	22
4	406.17	Y	2177.05	21
5		S	2013.99	20
6		I	1926.96	19
7	705.36	V	1813.872	18
8	820.38	D	1714.803	17
9	967.45	F	1599.776	16
10	1080.54	L	1452.708	15
11		I	1339.624	14
12		T	1226.54	13
13		A	1125.492	12
14		G	1054.455	11
15		A	997.4334	10
16		N	926.3962	9
17		V	812.3533	8
18		N	713.2849	7
19		S	599.242	6
20		P	512.21	5
21		D	415.1572	4
22		F	300.1302	3
23	2344.12	H	213.0982	2
24		G		1

iASPP-OH 24mer

#	b	Seq	y	#
1		G		24
2		A		23
3	243.11	N	2307.089	22
4	406.17	Y	2193.046	21
5	493.20	S	2029.983	20
6	606.29	I	1942.951	19
7	705.36	V	1829.866	18
8	820.38	D	1730.798	17
9	967.45	F	1615.771	16
10	1080.54	L	1468.703	15
11	1193.62	I	1355.619	14
12		T	1242.535	13
13		A	1141.487	12
14		G	1070.45	11
15		A	1013.428	10
16		N	942.3912	9
17		V	828.3482	8
18		N (OH)	729.2798	7
19		S	599.242	6
20		P	512.21	5
21		D	415.1572	4
22		F	300.1302	3
23	2360.12	H	213.0982	2
24		G		1

Table S2 Data collection and refinement statistics of FIH complexes with ASPP peptides substrates

**Datasets**	**FIH**.Zn(II).NOG iASPP	**FIH**.Zn(II).NOG ASPP1	**FIH**.Zn(II).NOG ASPP2
**PDB code**	**6HL6**	**6HL5**	**6HKP**
**Data Collection**			
Beamline (Wavelength, Å)	DLS I04-1 (0.9159)	DLS I02 (0.9795)	DLS I04-1 (0.9159)
Detector	Pilatus 6M-F	Pilatus 6M-F	Pilatus 6M-F
Data Processing	Xia2/ DIALS	Xia2/ DIALS	MOSFLM/SCALA
Space group	$P4_12_12$	$P4_12_12$	$P4_12_12$
Cell dimensions			
a,*b*,*c* (Å)	86.1, 86.1, 148.1	86.2, 86.2, 147.1	86.0, 86.0, 148.5
α,β,γ(°)	90, 90, 90	90, 90, 90	90, 90, 90
No. of molecules/ASU	1	1	1
No. reflections	40372 (1980)*	39456 (2880)*	44748 (6404)*
Resolution (Å)	74.51-1.97 (2.00-1.97)*	56.31-1.98 (2.03-1.98)*	86.02-1.90 (2.00-1.90)*
R_{merge}	0.079 (2.105)*	0.080 (2.35)*	0.180 (1.923)*
I/σI	14.8 (1.3)*	17.0 (1.4)*	8.3 (1.5)*
Completeness (%)	100 (98.9)*	100.0 (100.0)*	100 (100)*
Multiplicity	8.5 (7.8)*	18.9 (17.9)*	16.0 (15.6)*
Wilson B value (Å^2)	33.8	40.7	38.8
**Refinement**	PHENIX	PHENIX	PHENIX
R_{work}/R_{free}	0.1706/0.2055	0.1828/0.2013	0.1755/0.1948
No. atoms			
-Enzyme (A/B)	2711	2693	2709
-Ligand	NOG (10)	NOG (10)	NOG (10)
-Substrate	115	94	98
-Water	235	139	177
Avg. B-factors			
Enzyme	45.9	57.5	54.4
Ligand	32.0	42.5	38.8
Substrate	68.3	87.5	92.4
Water	49.7	35.5	58.1
R.m.s deviations			
-Bond lengths (Å)	0.011	0.014	0.005
-Bond angles (°)	0.956	1.150	0.684

* Highest resolution shell in parentheses.

[†]$R_{factor} = \Sigma_{hkl}||F_{obs}(hkl)|-k|F_{calc}(hkl)||/\Sigma_{hkl}|F_{obs}(hkl)|$, R_{free} is the R_{factor} for ~5%of reflections excluded from the refinement.

Table S3 Buffer and vapour diffusion conditions used for FIH complex crystallisation.

Protein complex	Sample composition	crystallisation conditions	Vapour diffusion conditions
**FIH** Zn(II)/NOG iASPP	11 mg/mL FIH (0.27 mM) in 50 mM Tris-HCl pH 7.5, peptide (1-2 mM) zinc acetate (0.5 mM)	0.1 M HEPES pH 7.5, 1.6 M ammonium sulfate, 3.5% PEG400	Sitting drop (300 nL) protein-to-well ratio 2:1, 293K
**FIH** Zn(II)/NOG ASPP1	11 mg/mL FIH (0.27 mM) in 50 mM Tris-HCl pH 7.5, peptide (1-2 mM) zinc acetate (0.5 mM)	0.1 M HEPES pH 7.5, 1.2 M ammonium sulfate, 4.5% PEG400	Sitting drop (300 nL) protein-to-well ratio 2:1, 293K
**FIH** Zn(II)/NOG ASPP2	11 mg/mL FIH (0.27 mM) in 50 mM Tris-HCl pH 7.5, peptide (1-2 mM) zinc acetate (0.5 mM)	0.1 M HEPES pH 7.5, 2.0 M ammonium sulfate, 5.5% PEG400	Sitting drop (300 nL) protein-to-well ratio 2:1, 293K

References

1. Hardy, A. P., Prokes, I., Kelly, L., Campbell, I. D., and Schofield, C. J. (2009) Asparaginyl beta-hydroxylation of proteins containing ankyrin repeat domains influences their stability and function. *J Mol Biol* **392**, 994-1006
2. Canning, P., von Delft, F., and Bullock, A. N. (2012) Structural basis for ASPP2 recognition by the tumor suppressor p73. *J Mol Biol* **423**, 515-527
3. Coleman, M. L., McDonough, M. A., Hewitson, K. S., Coles, C., Mecinovic, J., Edelmann, M., Cook, K. M., Cockman, M. E., Lancaster, D. E., Kessler, B. M., Oldham, N. J., Ratcliffe, P. J., and Schofield, C. J. (2007) Asparaginyl hydroxylation of the Notch ankyrin repeat domain by factor inhibiting hypoxia-inducible factor. *J Biol Chem* **282**, 24027-24038
